# Analytical characterization of volatiles present in the whole body odour of zebra finches

**DOI:** 10.1007/s00216-024-05466-8

**Published:** 2024-08-09

**Authors:** Tatjana Alves Soares, Barbara A. Caspers, Daniel Veit, Helene M. Loos

**Affiliations:** 1 Department of Chemistry and Pharmacy, Friedrich-Alexander-Universität Erlangen-Nürnberg (FAU), Henkestraße 9, 91054 Erlangen, Germany; 2 Department of Behavioural Ecology, Bielefeld University, Konsequenz 45, 33615 Bielefeld, Germany; 3 Joint Institute for Individualisation in a Changing Environment (JICE), University of Münster and Bielefeld University, Bielefeld, Germany; 4 Scientific Workshop, Max-Planck Institute for Chemical Ecology, Jena, Germany; 5 Fraunhofer Institute for Process Engineering and Packaging IVV, Giggenhauser Straße 35, 85354 Freising, Germany; 6 FAU Research Center “New Bioactive Compounds” (FAU NeW), Erlangen, Germany

**Keywords:** *Taeniopygia castanotis*, Open loop system, Chemical profile, Bird odour, Chemical sex signature, Gas chromatography–olfactometry

## Abstract

In recent decades, the compositions of preen oil and feathers have been studied to achieve insights into the chemistry of avian odours, which play a significant role in birds’ social behaviour. Fewer studies are available regarding volatiles originating from other sources, such as faeces, eggs or a bird’s whole body. The aims of this study were (i) to identify odour-active and further volatile compounds in zebra finch whole body odour and (ii) to semi-quantify selected volatiles and use the information to evaluate two different adsorbents for their suitability for whole body odour sampling. Volatiles from the headspace above zebra finches were sampled using an open loop system equipped with either activated charcoal or Tenax® TA. Samples were analysed by olfactory-guided approaches as well as gas chromatography–mass spectrometry. Using activated charcoal as sorbent, 26 odour-active and 73 further volatile compounds were detected, whereas with Tenax® TA 27 odour-active and 81 further volatile compounds were detected. In total, 104 compounds were (tentatively) identified, of which 22 had not been identified previously in zebra finch odour and 12 had not been described in any birds. Hints towards a chemical sex signature became evident for qualitative but not for quantitative differences. With the exception of some compounds, notably carboxylic acids and alkanes, relative peak areas obtained with the two adsorbent types were comparable. The approach described herein is proposed for future studies aiming to determine volatiles emitted by birds when, for example, parent birds are approaching the nest.

**Graphical Abstract:**

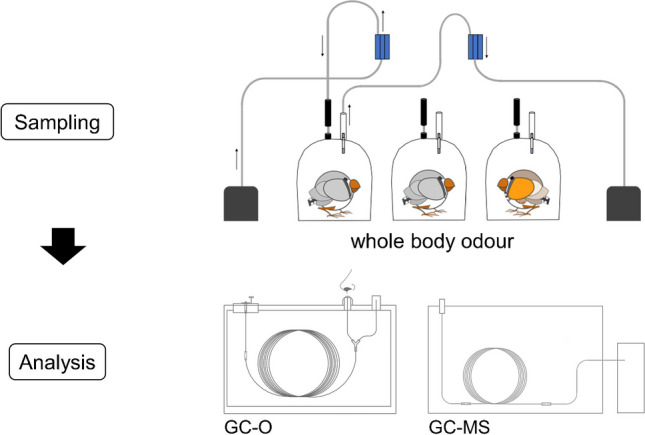

**Supplementary Information:**

The online version contains supplementary material available at 10.1007/s00216-024-05466-8.

## Introduction

Avian social olfaction has received increasing attention throughout recent decades. Researchers aimed to investigate the social contexts in which a bird’s body odour plays a role and also its composition [1–3]. For behavioural studies, researchers used (hidden) living birds [4, 5], eggs [6] or faeces [7] as a direct odour source or, indirectly, bird odour samples presented on materials such as cotton bags [8, 9], cotton swabs [10, 11], cotton balls [12] or nylon socks [13–15]. For chemical analysis, (the headspace above) preen oil, feathers from different body regions, faeces, eggs and the whole body has been investigated, mainly using solvent extraction [16–18], stir bar sorptive extraction (SBSE) [19–21] or solid-phase microextraction (SPME) [22–24], to extract the volatile compounds. Another option to extract volatiles from the headspace is to use adsorption tubes. Sorbent tubes have two advantages compared to SBSE and SPME: more types of sorbents are commercially available and their capacity is higher. In 2006, Douglas used this approach for the first time in the field of bird odour analysis [25]. Crested auklets were captured and placed into glass reaction kettles with a regulated airstream. Volatile emissions from these birds were successfully sampled onto polymer traps filled with Super Q or Tenax® TA. Since then, this principle has been used in six further studies on bird odour. Krause et al. (2014) trapped the volatile compounds of zebra finches and diamond firetails on nylon socks impregnated with the odour of individual birds onto activated charcoal [13]. Douglas and colleagues investigated octanal emissions from crested auklets using a glass reaction kettle and polymer traps [26, 27]. Diez-Fernandez and colleagues sampled the headspace of birds using glass desiccators and Tenax® TA cartridges to test the attraction of mosquitoes towards the odours of uninfected and *Plasmodium*-infected house sparrows [28]. Spanoudis et al. (2020, 2022) investigated the attraction of mosquitoes towards natural [29, 30] and synthetic chicken odour [30], as well as pigeon and magpie odour [29], sampled with Porapak® Q-packed adsorbent tubes. Sampling the volatile emissions of birds in this way has the advantage that the birds are not harmed [25]. Furthermore, contaminations from plants, insects and other naturally occurring materials can be excluded because the individual birds are isolated in chambers [25]. Additionally, sampling the whole body odour most closely reflects real-life situations, such as parental birds approaching the nest.

In the present study, our goal was to elucidate and semi-quantify volatiles emitted by zebra finches (*Taeniopygia castanotis*). With this aim, the whole body odour (for the sake of convenience herein defined as volatiles occurring in the headspace above a bird) of zebra finches was collected using an open loop system. The sorbents Tenax® TA 60/80 and activated charcoal were comparatively evaluated. We analysed the volatiles via one-dimensional gas chromatography–mass spectrometry (GC–MS) and additionally with olfaction-guided approaches, namely gas chromatography–flame ionization detection/olfactometry (GC-FID/O) and two-dimensional GC–MS/olfactometry (GC-GC–MS/O). These complementary techniques were used because odour-active substances are often only present in trace amounts and not detectable via classical GC–MS. For evaluation, we have split the volatile compounds into two groups, which we describe as ‘odour-active compounds’ and ‘further volatile compounds’. Odour-active compounds are defined herein as substances that are perceivable by the human nose during GC-FID/O analysis, and further volatile compounds are defined herein as compounds that were not perceived by the human nose during GC-FID/O but were detected via GC–MS. We compared the identified compounds with our previous results regarding volatiles present in preen oil and feathers [31]. Moreover, we evaluated whether a chemical sex signature would become evident.

## Materials and methods

### Chemicals

All chemicals used in this research are listed in the Supplementary Material.

### Animals and sampling

Samples were taken from a laboratory population of domesticated zebra finches (also called ‘DOM Bielefeld’ or ‘Bielefeld population’ [32, 33]) located in the Department of Animal Behaviour, Bielefeld University, Germany. All procedures with the zebra finches were approved by the German authorities (LANUV License Number AZ 81–02.04.2021.A432).

#### Sorbent preparation

Before sampling, activated charcoal tubes were prepared by passing 600 µl of each of the following solvents through the adsorbent: methanol, dichloromethane, chloroform and hexane. Afterwards, the tubes were baked at 150 °C for 1 h. Tenax® TA tubes were freshly packed each time and afterwards conditioned at 320 °C for 1 h under nitrogen flow using a tube conditioner (Gerstel GmbH & Co. KG, Mülheim an der Ruhr, Germany). After conditioning, the Tenax® TA and charcoal tubes were stored airtight sealed for a maximum period of 1 month until usage.

#### Headspace extraction

**Fig. 1:**
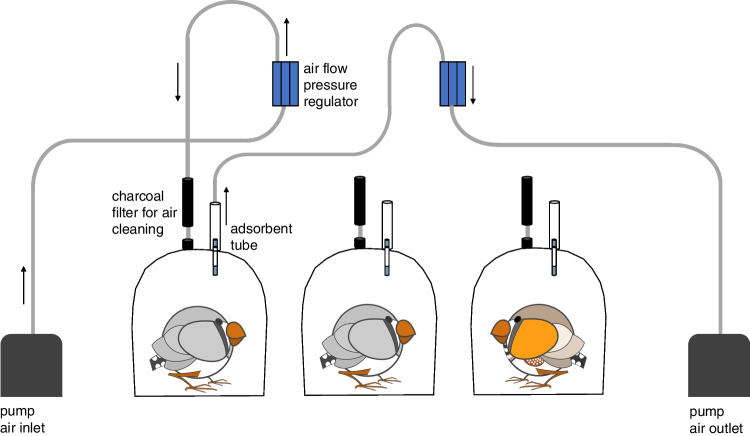
Setup of the open loop system used for the sampling of zebra finch whole body odour. For a better view of the air flow, only one chamber is connected in this figure

Whole body odour samples were collected by placing individual birds into glass cylinders (15 cm diameter, 15 cm height) that were closed towards the bottom and top with polytetrafluoroethylene disks, and volatiles of the headspace were sampled for 30 min via an open loop system [34]. The apparatus consisted of three separate cylinders so that three birds could be sampled at the same time (see Fig. 1). The outer sides of the cylinders were wrapped with towels but the interspaces between the cylinders were left unwrapped so that the birds could see each other and stay calm in the cylinder during the sampling procedure. A pump (Laboport N96, KNF, Freiburg, Germany) blew air into the cylinder at a flow rate of 0.7–0.8 l/min; the air was filtered through a charcoal filter (15 g) before entering the glass cylinder. A second pump sucked the air out of the cylinder at a flow rate of 0.4–0.5 l/min to guarantee that more air was entering than leaving the cylinder. The air that left the cylinder was passed through an adsorbent, whereby the volatile compounds were trapped onto the adsorbent, which was either activated charcoal (1.5 mg; Brechbuehler AG, Schlieren, Switzerland) or Tenax® TA 60/80 (200 mg). The tubes were then sealed with plastic caps and immediately frozen at − 20 °C until work-up for analysis, for a maximum period of 1 month.

#### Elution and concentration

After sampling, when preparing for analysis, the volatiles were eluted with 1 ml of dichloromethane and ethoxyethane for activated charcoal and Tenax® TA, respectively. The extracts of 10 samples (see Tables 1 and 2) were united in an Erlenmeyer flask and dried over anhydrous sodium sulfate. Afterwards, the extract was concentrated by means of Vigreux distillation and microdistillation to a volume of 100 µl at 50 °C. The concentrated extract was stored at − 80 °C until analysis.

**Table 1:** Overview of investigated sample pools for identification of odour-active and further volatile compounds

Name of pool	Sample type
10C	10 activated charcoal tubes with volatiles trapped from 5 different female and 5 different male birds
10BC1	10 activated charcoal tubes with volatiles trapped from empty sampling system
10T	10 Tenax® TA tubes with volatiles trapped from 5 different female and 5 different male birds
10BT1	10 Tenax® TA tubes with volatiles trapped from empty sampling system

**Table 2:** Overview of investigated sample pools for semi-quantification and identification of further volatile compounds

Name of pool	Sample type
10CF1, 10CF2, 10CF3	10 activated charcoal tubes with volatiles trapped from 10 different female birds
10CM1, 10CM2, 10CM3	10 activated charcoal tubes with volatiles trapped from 10 different male birds
10TF1, 10TF2, 10TF3	10 Tenax® TA tubes with volatiles trapped from 10 different female birds
10TM1, 10TM2, 10TM3	10 Tenax® TA tubes with volatiles trapped from 10 different male birds
10BC2, 10BC3, 10BT1, 10BT2	10 activated charcoal tubes or 10 Tenax® TA tubes with volatiles trapped from empty sampling system

#### Sample pools

For identification of odour-active and further volatile compounds, individual samples were pooled as described in Table 1. For semi-quantification and identification of further volatile compounds, additional sample pools were used (Table 2). Blank samples were prepared by pumping air through the empty system. The blank tubes were worked up analogously to the other samples.

### Gas chromatography–flame ionization detection/olfactometry and odour extract dilution analysis

For GC-FID/O, a Trace Ultra gas chromatograph from Thermo Fisher Scientific Inc. (Dreieich, Germany) was used, equipped with an uncoated, deactivated fused silica capillary (2.5–5 m length, 0.32 mm diameter) and either a DB-FFAP or DB-5 column (both 30 m length, 0.32 mm diameter, 0.25 µm film thickness; Agilent Technologies Inc., Santa Clara, CA, USA). Extracts (2 µl) were manually injected by using the cold-on-column technique with an injection temperature of 40 °C. After passing the pre-column and the main column, the eluent was split, with one part reaching the detector and the other part reaching the sniffing port. The oven temperature programme was as follows: start at 40 °C with a hold time of 2 min, followed by a temperature ramp of 10 °C/min until 240 °C on the DB-FFAP and 300 °C on the DB-5 column. The final temperature was held for 10 min. Helium was used as the carrier gas at a flow rate of 2.5 ml/min. The detector and sniffing port temperatures were 270 °C and 250 °C. For odour extract dilution analysis (OEDA), a DB-FFAP column was used. For identification, samples as well as standards were run on both a DB-FFAP and a DB-5 column. An OEDA was performed with one sample pool for each adsorbent type (see Table 1), with the aim of ranking the odour compounds according to their potential contribution to the overall odour. For OEDA, the distillates were diluted step-by-step in a ratio of 1:1 with dichloromethane or ethoxyethane, and afterwards 2 µl of each dilution step was analysed by a trained panelist until no compounds were perceivable at the sniffing port. The odour dilution (OD) factor resembling the last dilution in which an odour was perceivable was noted for every odour impression. Blank samples (Table 1) were analysed analogously to the other sample pools.

### Gas chromatography–mass spectrometry

GC–MS measurements for identification and semi-quantification were obtained using a GC 7890A and an MSD 5975C (Agilent Technologies Inc., Santa Clara, CA, USA) equipped with an MPS 2 XL autosampler and a CIS4 injection system from Gerstel GmbH & Co. KG (Mülheim an der Ruhr, Germany). The system was equipped with an uncoated, deactivated fused silica capillary (2.5–5 m length, 0.53 mm diameter) serving as a pre-column and either a DB-FFAP or DB-5 column (both 30 m length, 0.25 mm diameter, 0.25 µm film thickness) from Agilent Technologies Inc. (Santa Clara, CA, USA). The main column was connected to the detector through an uncoated fused silica capillary (0.3–1 m length, 0.25 mm diameter) and helium was used as the carrier gas at a flow rate of 1.0 ml/min. Mass spectra were recorded in electron ionization (EI) and total ion current (TIC) mode with a mass to charge ratio (*m*/*z*) of 40–400 and ionization energy of 70 eV. The oven temperature programme was as follows: start at 40 °C for 5 min; temperature ramp at 10 °C/min; final temperature 240 °C (hold time 10 min). The injection volume for each run was 1 µl and injection was carried out on-column.

### Heart-cut two-dimensional gas chromatography–mass spectrometry/olfactometry

Additional analysis was performed with a heart-cut two-dimensional GC–MS/O system. This system was equipped with two GC 7890B chromatographs coupled to an MS 5977B (all from Agilent Technologies Inc., Santa Clara, CA, USA). The injection (1 µl, cold on-column) was carried out via an MPS 2 autosampler from Gerstel GmbH & Co. KG (Mülheim an der Ruhr, Germany). The first oven was equipped with a DB-FFAP column (30 m length, 0.32 mm diameter, 0.25 µm film thickness; Agilent Technologies Inc., Santa Clara, CA, USA), an MCS 2 multi-column switching system (Gerstel GmbH & Co. KG, Mülheim an der Ruhr, Germany) and a flame ionization detector (FID); both ovens were connected through a cryo-trap system (CTS1; Gerstel GmbH & Co. KG, Mülheim an der Ruhr, Germany). In the second oven, a DB-5 column (30 m length, 0.25 mm diameter, 0.25 µm film thickness; Agilent Technologies Inc., Santa Clara, CA, USA) was installed. The carrier gas was helium, at a flow rate of 2.5 ml/min. In the first oven, the sample was split into two parts. One reached the CTS1 and the other was split between the FID detector and the odour detection port (ODP3; Gerstel GmbH & Co. KG, Mülheim an der Ruhr, Germany). Behind the CTS1, the cut part of the sample was transferred onto the second main column, which was directly connected to the MS detector and an ODP. All connecting columns, as well as the pre-column in the first oven, were made of uncoated, deactivated fused silica. The temperature of the FID detector was 280 °C and the ODPs were heated to 250 °C. EI mass spectra were recorded in scan mode (40–400 m*/z*) with an ionization energy of 70 eV. The temperature programme started at 40 °C (hold time 2 min for the first oven and 1 min for the second oven), followed by a temperature ramp of 8 °C/min until reaching the final temperature of 240 °C (first oven) or 250 °C (second oven). The final temperatures were held for 5 min.

### Identification criteria

The identification of odour-active substances was achieved by comparing odour qualities, retention indices (RI) and mass spectra with those of known reference compounds on two columns of different polarity [35]. For the identification of further odourless volatiles, retention indices and mass spectra were compared with data from reference compounds obtained on two columns of different polarity, applying an in-house database established using AMDIS software (version 2.72; National Institute for Standards and Technology, Gaithersburg, USA). Criteria for RI matches were ± 20 for DB-FFAP measurements and ± 10 for DB-5 measurements. The criterion for MS matches was a match of ≥ 80. Identification is considered tentative in cases where this comparison was achieved only on one column or where no mass spectrum was obtained for an odour-active compound (see also Tables S1 and S2, Supplementary Material). RIs were calculated through the measurement of a series of homologous alkanes (C6–C30).

### Semi-quantification of selected volatiles and comparison of sorbent types

For semi-quantification, 50 µl of a 50 µg/ml methyl octanoate solution was added to each sample pool after elution from the sorbent tubes before distillation. Relative peak areas for the analytes were obtained by dividing the area of the respective substance by the area of the internal standard, multiplying by 1000 for better readability. Depending on the properties and detection of a substance, evaluation on either a DB-FFAP or DB-5 column was selected. Areas were obtained by automatic integration of the AMDIS software. A substance was semi-quantified if it was identified in at least three of six samples of i) both activated charcoal and Tenax® TA samples or ii) both male and female samples (see Table 2). Additionally, if a substance was present only in one adsorbent type, a substance was semi-quantified if it was present in at least two of three samples of both sexes.

### Statistical analysis

Data were analysed using SPSS Statistics (version 29.0.1.0; IBM Corp., Armonk, NY, USA). Two repeated-measures ANOVAs were calculated: for answering the research question of whether there is a significant difference between activated charcoal and Tenax® TA as a sorbent medium, ANOVA I was calculated using substance (48), adsorbent (2) and sex (2) as independent factors; and to answer the research question of whether there is a significant difference between male and female zebra finches, ANOVA II was calculated using substance (57), adsorbent (2) and sex (2) as independent factors. For both ANOVAs, substances were included if a substance was identified in at least three of six samples of each group (activated charcoal and Tenax® TA for ANOVA I and female and male for ANOVA II; see Table S3). For ANOVA II, substances were additionally included if they were present only in one adsorbent type and, if so, in two out of three samples of each sex. Tables S4 and S5 in the Supplementary Material give the results for ANOVAs I and II. Greenhouse–Geisser adjustment was used for correction of sphericity and Bonferroni-adjusted post hoc analysis was performed for the identification of significant differences between groups.

## Results

Sampling of whole body odour was performed using two different adsorbents: activated charcoal and Tenax® TA. The results for identification of compounds, semi-quantification of selected compounds and differences between adsorbent types and sexes are addressed in turn.

### Identification

We used two different approaches for the identification of volatiles in the whole body odour samples: odour-active compounds were identified in two sample pools and two blank pools (Table 1) using GC-FID/O, GC–MS and GC-GC–MS/O; and further volatile compounds were identified in fourteen sample pools and five blank pools (Tables 1 and 2) using GC–MS. This approach was chosen because odour-active compounds often occur in trace amounts and therefore cannot be detected accurately via classical GC–MS approaches. As we do not know which compounds are odour-active for birds, we aimed to comprehensively characterize the volatilome via these complementary methods.

### Odour-active compounds in whole body odour samples

Using olfaction-guided approaches, 26 and 27 odour-active substances were detected in the sample pools obtained with activated charcoal and Tenax® TA, respectively. Among these substances, 4 and 3 substances, respectively, were identified and 17 and 14, respectively, were tentatively identified; 5 and 10 substances, respectively, remained unknown. Twenty-five of these compounds were detected in at least one of the samples but not in the blanks. These compounds were: the mushroom-like smelling oct-1-en-3-one; the aldehydes (*Z*)-non-2-enal (fatty, soapy, cucumber-like), (*E*)-non-2-enal (fatty, cucumber-like, cardboard-like), (*E,E*)-2,4-nonadienal (fatty, nutty), (*E,E*)-2,4-decadienal (fatty, deep-fried) and trans-4,5-epoxy-(*E*)-2-decenal (metallic); the acids 2/3-methylbutanoic acid (co-eluting, apple-like, fruity/cheesy) and 4-ethyloctanoic/4-methylnonanoic acid (co-eluting, goat-like/cardboard-like, plastic-like); geosmin (earthy, mouldy); 2-methoxyphenol (smoky, smoked ham-like); *γ*-nonalactone (coconut-like); and several unknown substances. For activated charcoal, the highest OD factor (128) was determined for 1,3-benzothiazole; for Tenax® TA, the highest OD factors (16384 each) were found for 3-/2-methylbutanoic acid (co-eluting), (*Z*)-2-butyloct-2-enal and unknown compound no. 18 (Table 3; for further information, see Table S1).

**Table 3:** Odour-active substances in zebra finch whole body odour samples 10C and 10t (see Table 1)

No	Substance	CAS no	RI DB-FFAP	RI DB-5	Odour quality	10C	10T	Previously identified?
1	Oct-1-en-3-one	4312–99-6	1291	979	Mushroom-like	RI, O, RC	RI, O, RC	y
2	Acetic acid^B^	64–19-7	1438	s.d	Vinegar-like	RI, O, MS, RC	RI, O, MS, RC	y
3	Unknown	-	1450	-	Cardboard-like		X	
4	(*Z*)-Non-2-enal	60784–31-8	1494	1145	Fatty, soapy, cucumber-like	RI, O, RC	RI, O, RC	y
5	**Benzaldehyde**^B^	100–52-7	1515	967	Bitter almond-like, almond-like	RI, O, MS, RC	RI, MS, RC	y
6	(*E*)-Non-2-enal	18829–56-6	1533	1160	Fatty, cucumber-like, cardboard-like	RI, O, RC		y
7	Unknown	-	1600	-	Green, fatty	X		
8	**Butanoic acid**^B^	107–92-6	1618	804	Cheesy, sweaty	RI, O, MS, RC	RI, MS, RC	y
9	3-/2-Methyl-butanoic acid*	503–74-2/116–53-0	1653/1655	861/868	Cheesy/apple-like, fruity	RI, O, MS, RC	RI, O, RC	y
10	(*Z*)-2-Butyloct-2-enal^B^	99915–14-7	1663	1373	Fruity	RI, O, RC	RI, O, RC	y
11	(*E,E*)-2,4-Nonadienal	5910–87-2	1690	1213	Fatty, nutty	RI, O, RC	RI, O, RC	y
12	Pentanoic acid^B^	109–52-4	1725	888	Fruity, sweaty, pungent	RI, O, MS, RC	RI, MS, RC	y
13	(*E,E*)-2,4-Decadienal	25152–84-5	1801	1327	Fatty, deep-fried	RI, O, RC	RI, O, RC	y
14	Geosmin	19700–21-1	1806	1421	Earthy, mouldy	RI, O, RC		y
15	2-Methoxyphenol	90–05-1	1846	1089	Smoky, smoked ham-like	RI, O, RC	RI, O, RC	
16	**1,3-Benzothiazole**^B^	95–16-9	1937	1227	Rubber-like, car tyre-like	RI, O, MS, RC	RI, MS, RC	y
17	**2-Methylsulfanyl-1,3-benzothiazole**^B^	615–22-5	1947	1235	Medicinal, smoky, phenolic	RI, O, MS, RC		
18	Unknown	-	1975	-	Car tyre-like, burnt		X	
19	*trans*-4,5-Epoxy-(*E*)-2-decenal	134454–31-2	1991	1375	Metallic	RI, O, RC	RI, O, RC	y
20	**ɣ-Nonalactone**	104–61-0	2018	1360	Coconut-like	RI, O, RC	RI, O, MS, RC	y
21	Unknown	-	2070	-	Mouldy, horse stable-like		X	
22	4-Ethyloctanoic acid/4-Methylnonanoic acid*	16493–80-4/54947–74-9	2187/2198	1322/1328	Goat-like/cardboard-like, plastic-like	RI, O, RC	RI, O, RC	y
23	Decanoic acid^B^	334–48-5	2267	1371	Coriander-like, plastic-like, soapy	RI, O, MS, RC	RI, O, MS, RC	y
24	Unknown	-	2318	-	Fatty, cardboard-like	X	X	
25	Unknown	-	2350	-	Fatty		X	
26	Unknown	-	2421	-	Citrus-like, coriander-like, waxy		X	
27	Unknown	-	2444	-	Eucalyptus-like, coriander-like	X		
28	Dodecanoic acid^B^	143–07-7	2473	1571	Waxy, soapy	RI, O, MS, RC	RI, MS, RC	y
29	Unknown	-	2521	-	Waxy		X	
30	Vanillin	121–33-5	2564	1399	Vanilla-like, sweet	RI, O, RC		y
31	Unknown	-	2581	-	Soapy, coriander-like	X		
32	Unknown	-	2589	-	Vanilla-like, cinnamon-like, green		X	
33	Unknown	-	2611	-	Vanilla-like		X	
34	Unknown	-	2633	-	Cheesy, mouldy	X		
35	Unknown	-	2674	-	Cheesy, honey-like		X	

The table shows the identified odour-active substances together with their retention indices (RI) on a DB-FFAP and a DB-5 column, their CAS number, their odour quality according to an in-house flavour language, identification criteria and previous reports in birds. Substances that occurred in odour dilution factors of < 4 are not shown. Identified substances (see the “Materials and methods” section) are marked in bold; tentatively identified substances are not in bold. Identification criteria: *O*, odour quality at sniffing port; *MS*, mass spectrum; *RI*, retention indices from internal database established with reference compounds; *RC*, comparison of respective data with reference compounds; *X*, unknown substance detected; *y*, reported in previous publications on bird odour. *Co-elution of two compounds; ^B^potential contaminant of exogenous origin because also detected in the blank sample (with MS and/or odour)

### Further volatile compounds in whole body odour

Further volatile compounds were identified with a focus on alcohols, aldehydes, alkanes, carboxylic acids, ketones and esters. In total, 73 and 81 compounds were detected in the whole body odour of zebra finches, sampled with activated charcoal and Tenax® TA, respectively. Of these, 29 and 22 compounds were identified, 38 and 49 were tentatively identified and 6 and 10 remained unknown, respectively. Thirteen substances were detected in at least one of the samples but not in the blanks. These substances were camphene, α-phellandrene, butyl acrylate, (*E*)-cinnamaldehyde, pentadecan-1-ol, 2-(2-ethoxyethoxy)ethanol, 3-methylbutanoic acid, oleic acid and five unknown compounds (RI: 1774, 2321, 2384, 2817, 2852). The identified substances together with their CAS numbers, RIs, identification criteria and previous identifications can be found in Table 4 (for more details, see Table S2).

**Table 4:** Further volatile compounds in zebra finch whole body odour samples

No	Name	CAS	RI DB-FFAP	RI DB-5	Activated charcoal	Tenax® TA	Previously identified?
1	Heptane^B^	142–82-5	700	700		t.i	
2	α-Pinene^B^	80–56-8	806	937	t.i	t.i	y
3	Nonane^B^	111–84-2	900	900	t.i		y
4	Decane^B^	124–18-5	1000	1000	t.i	t.i	y
5	4-Butylphenol^B^	1638–22-8	1033	1361	t.i		y
6	Camphene	79–92-5	1067	n.a	t.i		
7	**Hexanal**^**B**^	66–25-1	1080	800	i	i	y
8	Unknown^B^	-	1089	n.a	X		
9	Undecane^B^	1120–21-4	1100	1100	t.i	t.i	y
10	**1,4-/1,3-Xylene**^B*^	106–42-3/108–38-3	1130/1139	877/875	i	i	y
11	3-Carene^B^	13466–78-9	1140	n.a		t.i	y
12	Butan-1-ol^B^	71–36-3	1141	s.d	t.i	t.i	y
13	α-Phellandrene	99–83-2	1141	n.a		t.i	
14	**Heptan-2-one**^**B**^	110–43-0	1177	892		i	y
15	Butyl acrylate	141–32-2	1178	900	t.i		y
16	1,2-Xylene^B^	95–47-6	1179	899	t.i	t.i	y
17	Heptanal^B^	111–71-7	1179	906	t.i	t.i	y
18	**Dipentene**^**B**^	138–86-3	1192	1034	i	t.i	y
19	Dodecane^B^	112–40-3	1200	1200	t.i	t.i	y
20	**1-Ethyl-3-methylbenzene**^**B**^	620–14-4	1219	963	i		
21	**Pentan-1-ol**^**B**^	71–41-0	1236	776	t.i	i	y
22	**1-Ethyl-2-methylbenzene**^**B**^	611–14-3	1257	980	i	i	
23	***p*-/m-Cymene**^B*^	99–87-6/535–77-3	1262/1262	1027/1021	i	i	y
24	**Octanal**^**B**^	124–13-0	1280	1002	i	i	y
25	Tridecane^B^	629–50-5	1300	1300		t.i	y
26	**Hexan-1-ol**^**B**^	111–27-3	1336	872	i	i	y
27	**Nonanal**^**B**^	124–19-6	1381	1106	i	i	y
28	Tetradecane^B^	629–59-4	1400	1400	t.i	t.i	y
29	Heptan-1-ol^B^	111–70-6	1444	972	t.i	t.i	y
30	**Furan-2-carbaldehyde**^**B**^	98–01-1	1465	838	i	t.i	y
31	**2-Ethylhexan-1-ol**^**B**^	104–76-7	1468	1030	i	i	y
32	**Decanal**^**B**^	112–31-2	1483	1208	i	i	y
33	**Propanoic acid**^**B**^	79–09-4	1522	731	i	t.i	y
34	**Octan-1-ol**^**B**^	111–87-5	1545	1073	i	t.i	y
35	2-Methylpropanoic acid/ 2-Methylpropanal^B*^	79–31-2	1550/1561	779/n.a	t.i	t.i	y
36	**Undecanal**^**B**^	112–44-7	1592	1309		i	y
37	**Benzonitrile**^**B**^	100–47-0	1597	989	i	i	
38	Hexadecane^B^	544–76-3	1600	1600		t.i	y
39	**1-Phenylethanone**^**B**^	98–86-2	1613	1070	i	i	y
40	2-(2-Ethoxyethoxy)ethanol	111–90-0	1618	n.a		t.i	y
41	**Oxolan-2-one**^**B**^	96–48-0	1626	915		i	
42	Nonan-1-ol^B^	143–08-8	1637	1173	t.i	t.i	y
43	Dodecanal^B^	112–54-9	1698	1411		t.i	y
44	Unknown^B^	-	1734	n.a		X	
45	Decan-1-ol^B^	112–30-1	1740	1274	t.i	t.i	y
46	**1-(3-Methylphenyl)ethanone**^**B**^	585–74-0	1745	1175	i		
47	**Dioctyl ether**^**B**^	629–82-3	1745	1664	i		y
48	**1-(4-Methylphenyl)ethanone**^**B**^	122–00-9	1766	1188	i		
49	Unknown	-	1774	n.a		X	
50	Octadecane^B^	593–45-3	1800	1800		t.i	y
51	**Hexanoic acid**^**B**^	142–62-1	1827	993	i	t.i	y
52	Unknown^B^	-	1854	n.a		X	
53	Phenylmethanol^B^	100–51-6	1866	1038	t.i	t.i	y
54	Unknown^B^	-	1894	-		X	
55	Heptanoic acid^B^	111–14-8	1934	1086	t.i	t.i	y
56	**Dodecan-1-ol**^**B**^	112–53-8	1946	1476	i	t.i	y
57	Icosane^B^	112–95-8	2000	2000		t.i	y
58	**Phenol**^**B**^	108–95-2	2000	981	i	i	y
59	Isopropyl myristate^B^	110–27-0	2025	1823	t.i	t.i	y
60	(*E*)-Cinnamaldehyde	104–55-2	2035	1276	t.i		
61	Octanoic acid^B^	124–07-2	2052	1179	t.i	t.i	y
62	(*Z*)-Hex-3-enal^B^	6789–80-6	2071	806	t.i		y
63	Henicosane^B^	629–94-7	2100	2100	t.i		y
64	**Nonanoic acid**^**B**^	112–05-0	2149	1270	i	t.i	y
65	**Tetradecan-1-ol**^**B**^	112–72-1	2153	1679	i	t.i	y
66	Docosane^B^	629–97-0	2200	2200	t.i	t.i	y
67	**Methyl hexadecanoate**^**B**^	112–39-0	2203	1924	i	i	y
68	Isopropyl palmitate^B^	142–91-6	2230	2022	t.i	t.i	y
69	Unknown^B^	-	2246	n.a		X	
70	Pentadecan-1-ol	629–76-5	2261	1782		t.i	y
71	Unknown^B^	-	2270	n.a		X	
72	Tricosane^B^	638–67-5	2300	2300	t.i	t.i	y
73	**2,4-di-*tert*-Butylphenol**^**B**^	96–76-4	2306	1509		i	y
74	Unknown	-	2321	n.a		X	
75	Hexadecan-1-ol^B^	36653–82-4	2362	1896	t.i	t.i	y
76	Unknown	-	2384	n.a	X		
77	Tetracosane^B^	646–31-1	2400	2400	t.i	t.i	y
78	**Methyl octadecanoate**^**B**^	112–61-8	2413	2126	i	i	y
79	Unknown^B^	-	2460	n.a	X		
80	Diphenylmethanone^B^	119–61-9	2477	n.a	t.i	t.i	y
81	Pentacosane^B^	629–99-2	2500	2500	t.i	t.i	
82	**Octadecan-1-ol**^**B**^	112–92-5	2577	2089	i	i	y
83	Hexacosane^B^	630–01-3	2600	2600	t.i	t.i	y
84	Unknown^B^	-	2643	n.a		X	
85	Unknown^B^	-	2658	n.a	X		
86	Tetradecanoic acid^B^	39525–69-4	2682	1761	t.i	t.i	y
87	Heptacosane^B^	593–49-7	2700	2700	t.i	t.i	y
88	Unknown^B^	-	2754	n.a	X		
89	Unknown^B^	-	2770	n.a		X	
90	Octacosane^B^	630–02-4	2800	2800	t.i	t.i	y
91	Unknown	-	2817	n.a		X	
92	Unknown	-	2852	n.a	X		
93	**Hexadecanoic acid**^**B**^	57–10-3	2895	1960	i	i	y
94	Nonacosane^B^	630–03-5	2900	2900	t.i	t.i	y
95	**Docosan-1-ol**^**B**^	661–19-8	2996	2497	i		y
96	Triacontane^B^	638–68-6	3000	3000	t.i	t.i	y
97	**Octadecanoic acid**^**B**^	57–11-4	3135	2162		i	y
98	Oleic acid	112–80-1	3167	2137		t.i	y
99	Benzene-1,2-dicarboxylate (in the following DEHP)^B^	117–81-7	3201	n.a	t.i	t.i	y

The table shows retention indices (RI) on a DB-FFAP and a DB-5 column, CAS-numbers, fulfilled identification criteria and previous reports. Identified compounds are marked in bold. *RI*, retention indices from internal database established with reference compounds; *i*, identified by comparison of RI and match with a reference compound on two columns (DB-FFAP, DB-5) via AMDIS; *t.i.*, tentatively identified by comparison of RI and match with a reference compound on a DB-FFAP or a DB-5 column via AMDIS; *n.a.*, not available; *s.d.*, elution within solvent delay; ^B^Potential contaminant of exogenous origin because substance was also present in the blank sample; *co-elution of two compounds; *X*, unknown compound detected. Substances that have been reported before in bird odour are marked with a ‘y’ in the far right column. For each adsorbent type, a substance is mentioned as (tentatively) identified if it was detected in > 2 samples

### Semi-quantification of selected volatiles

Substances were semi-quantified if a substance was detected in at least three out of six samples of both activated charcoal and Tenax® TA samples or both male and female samples (see Table 2). Only identified substances were semi-quantified (for an overview of the semi-quantified substances, see also Table S3). In both datasets, overall average relative peak areas ranged from 0.8 ± 1.5 [× 10^−3^] (hexan-1-ol – Tenax® TA – male) to 3719.7 ± 963.5 [× 10^−3^] (DEHP – activated charcoal – female). Table S3 shows the average relative peak areas together with their standard deviations (SD) for female and male whole body odour sampled with Tenax® TA and activated charcoal, together with the relative peak areas obtained for the blank samples.

### Impact of adsorbent type

For evaluation of the impact of adsorbent type on the composition of the eluates, qualitative and quantitative differences were assessed. Qualitative comparison of all detected substances showed that 17 odour-active substances and one unknown compound (RI = 2318) were common to samples from both sorbent media and that four substances ((*E*)-non-2-enal, geosmin, 2-methylsulfanyl-1,3-benzothiazole and vanillin) and four unknown compounds were exclusively identified in samples obtained with activated charcoal (see also Table 3). Nine unknown compounds were exclusively detected in the Tenax® TA samples. In terms of further volatile compounds, samples obtained with activated charcoal and Tenax® TA had 55 compounds in common and 12 and 16 compounds were exclusively identified in activated charcoal and Tenax® TA, respectively (see Table 4). Six and ten unknown compounds were detected only in activated charcoal and Tenax® TA samples, respectively.

**Fig. 2:**
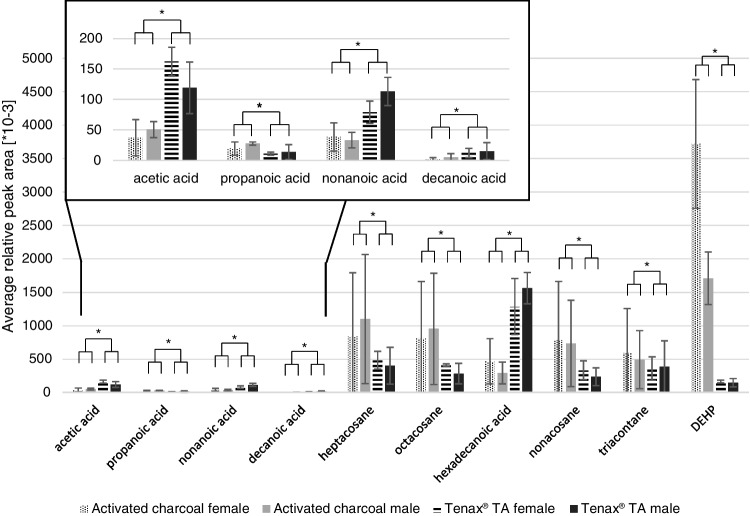
Average relative peak areas [× 10^−3^] (± SD; *n* = 3 for each group) of volatiles that differed significantly between activated charcoal and Tenax ® TA. *Significant difference

The quantitative impact of adsorbent type was assessed by statistically analysing the relative peak areas of the semi-quantified substances (Table S3). ANOVA I showed that there was no significant effect of the adsorbent [*F*(1.000, 2.000) = 2.812, *p* = 0.236]. However, the interaction of substance and adsorbent revealed a significant effect [*F*(1.650, 3.299) = 22.785, *p* < 0.012, partial *η*^2^ = 0.919]. Post hoc analysis showed that for four carboxylic acids (acetic, nonanoic, decanoic and hexadecanoic acid), the relative peak areas were significantly higher whereas for propanoic acid, DEHP and four alkanes (heptacosane, octacosane, nonacosane and triacontane), they were significantly lower in Tenax® TA compared to activated charcoal samples (see Fig. 2 and Table S3).

The standard deviations are given in Table S3. Additionally, we calculated the average and median relative standard deviation (RSD) for each adsorbent (Table 5). For some substances, the average RSD values were acceptable, whereas for other substances they were quite high. The high variability of RSD was present in both the blank and bird samples.

**Table 5:** Average, range and median relative standard deviation (RSD) for activated charcoal and Tenax® TA tubes

Adsorbent type	Activated charcoal			Tenax® TA		
Sample type	Blank	Female	Male	Blank	Female	Male
Average RSD [%]	44.6	72.7	71.7	121.9	56.5	54.8
RSD range [%]	1.7–144.4	10.3–181.8	0.9–175.0	1.5–150.0	2.3–181.8	12.9–187.5
Median RSD [%]	72.2	94.4	87.5	125.0	75.1	84.2

### Difference between sexes

In a further step, we evaluated whether some compounds were exclusively detected in one sex. First, the dataset was screened for compounds that were detected in at least two samples of one sex and in no sample of the other sex to determine potential qualitative sex differences. Six compounds for activated charcoal and four for Tenax® TA fulfilled these criteria. Pentan-1-ol and octanoic acid were detected in male but not female activated charcoal samples, whereas in the case of Tenax® TA these compounds were only detected in samples from female zebra finches. Isopropyl myristate was only detected in male Tenax® TA samples and decan-1-ol only in female Tenax® TA samples, whereas both compounds were detected in both sexes in activated charcoal samples. Heptanoic acid and diphenylmethanone were detected only in activated charcoal samples from female and male birds, respectively, whereas in Tenax® TA samples, these substances were detected for both sexes. Two further compounds were found in only one sex in activated charcoal samples and were not detectable in the Tenax® TA samples. These compounds were 2-methylsulfanyl-1,3-benzothiazole in the male activated charcoal samples and (*E*)-cinnamaldehyde in the female activated charcoal samples. There was no substance for which consistent evidence regarding a potential sex signature was obtained across both adsorbent types.

The quantitative difference between semi-quantified compounds occurring in both male and female samples was evaluated using ANOVA II (Table S5), finding no significant main effect of sex [*F*(1.000, 2.000) = 0.047, *p* = 0.848] or the interaction of substance and sex [*F*(1.157, 2.315) = 1.065, *p* = 0.414].

## Discussion

In this study, we characterized the volatile composition of whole body odour of zebra finches. Both odour-active and further volatile compounds were investigated. In total, 104 compounds were identified in zebra finch whole body odour samples.

### Comparison with previous insights from zebra finch preen oil and feathers

**Fig. 3:**
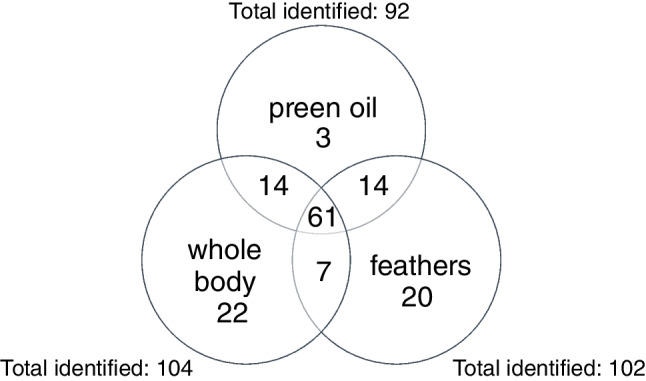
Numbers of compounds identified in zebra finch whole body odour samples, preen oil and feathers. Data obtained from this publication and from Alves Soares et al. [31]

Comparing the results with our previous study on zebra finch preen oil and feathers, where solvent extraction was used [31], 61 of the 104 substances identified here were detected in all three sample types (see also Fig. 3). Additionally, 14 further substances were detected in preen oil and whole body odour samples and another 7 substances were shared between feathers and whole body odour samples. Twenty-two compounds were only identified in whole body odour: the alkanes heptane, nonane, decane, tridecane, henicosane and pentacosane; the aldehydes dodecanal and (*E*)-cinnamaldehyde; the terpenes α-pinene, camphene and α-phellandrene; the aromatic compounds 1-ethyl-3-methylbenzene, 1-ethyl-2-methylbenzene, *p*-/*m*-cymene(co-eluting), benzonitrile, 2-methoxyphenol, 2-methylsulfanyl-1,3-benzothiazole, 1-(3-methylphenyl)ethanone, 1-(4-methylphenyl)ethanone and 1-phenylethanone; the lactone oxolan-2-one; and the carboxylic acid oleic acid. Twelve of the 22 compounds identified exclusively in whole body odour were also identified for the first time in bird odour. Compounds detected exclusively in whole body odour could either originate from body parts not sampled with feathers and preen oil (e.g., skin, eyes, breath, other excretions or faeces) or be detected due to the different analytical methods applied, which might come along with different sensitivities or artefacts.

In general, it is striking that most of the further volatile compounds were not only detected in the sample pools but also in the blank pools, and that high (relative) standard deviations occurred for some substances (Table 5, Table S3). Also, 10 of 21 (tentatively) identified odour-active compounds (Table S1) and 85 of 93 (tentatively) identified further volatile compounds (Table S2) were detected in the blanks. For future studies, a reduction in the background contamination is crucial and the reproducibility of the method should be checked for analytes of interest. To achieve this, the potential sources of the detected volatiles must be identified, which could be related to storage of the sorbent tubes, the open loop system and its materials, as well as cleaning procedures, or the sorbent material itself. Benzaldehyde, 1-phenylethanone, acetic acid, hexanal, heptanal, nonanal, decanal, phenol, benzonitrile and diphenylmethanone have previously been reported as artefacts in analyses using Tenax® TA adsorption tubes [36–40]. Furthermore, heptane, nonane and decane have been identified in insufficiently pre-conditioned Tenax® TA tubes [41]. Some of these substances, such as the aldehydes (known lipid oxidation products), might be of both exogenous and endogenous origin. Quantitative approaches therefore should be used in future studies, carefully evaluating the background levels of such substances. Additionally, preliminary trials should be performed to localize and prevent contamination of whole body odour samples by possible exogenous sources of volatile substances.

### Impact of adsorbent type

Two types of adsorbents were used to sample whole body odour samples: activated charcoal and Tenax® TA. Overall, similar results were obtained with both adsorbents. Only some substances were detected in significantly different amounts. Four carboxylic acids had significantly higher and four long-chain alkanes had significantly lower relative peak areas when using Tenax® TA compared to activated charcoal. The relative humidity of the sampled air influences the capacity of sorbent materials, depending on their structure and polarity [42–44]. It might be speculated that the differences in the relative peak areas of the above-mentioned substances could be related to the different impact of relative humidity on the capacity of the two sorbents [45, 46]. This should be experimentally determined in future studies and, in general, relative humidity should be controlled for in the study setups.

In previous studies, no major differences between the two adsorbent types were found. Baimatova et al. (2016) found only slight differences in the adsorption rate of benzene, toluene, ethylbenzene and 1,2-xylene [47]. The biggest difference was obtained for benzene, with activated charcoal completely adsorbing benzene and Tenax® TA adsorbing 77–79% [47]. Fischer et al. (2005) compared measurement techniques for the determination of microbial volatile organic compounds in indoor rooms [48]. The techniques of Tenax ® TA adsorption–thermal desorption and activated charcoal adsorption–elution led to similar results insofar the procedure for calibration was standardized [48]. Thus, from the analytical perspective, both adsorbent types appear suitable for the analysis of whole body odour. However, it should be noted that in terms of economic efficiency, activated charcoal tubes are cheaper in the long run because preliminary tests have shown that Tenax® TA tubes can be used only once in combination with solvent elution whereas activated charcoal tubes can be reconditioned. Indeed, Tenax® TA is regularly used for thermal desorption whereas charcoal is mainly used with liquid desorption. Therefore, the costs of subsequent analyses must also be considered. In addition, the option of trapping part of the thermally desorbed volatiles to allow for multiple injections of the same sample also after thermal desorption might prove useful in future analyses.

### Impact of the bird’s sex on volatile composition

Possible sex differences were evaluated in both a qualitative and quantitative manner, with seven (activated charcoal) and four (Tenax® TA) compounds suggested as potential candidates for a qualitative sex signature. These candidates included linear alcohols, carboxylic acids, two aromatic compounds and one sulfide. However, most of these compounds were detected in the other sex or in both sexes when considering the respective other sorbent type. Three substances occurred in all three samples of one sex and in no sample of the other sex: heptanoic acid and 2-methylsulfanyl-1,3-benzothiazole were detected in male activated charcoal samples and isopropyl myristate in female Tenax® TA samples. However, heptanoic acid and isopropyl myristate were detected in both sexes when applying the other adsorbent type. Only 2-methylsulfanyl-1,3-benzothiazole, not described in bird odour before, was detected in male activated charcoal samples and in no sample of the other sorbent type. However, this compound is used in consumer and industrial products, for example textiles and rubber, and for various applications, such as rubber vulcanization or corrosion inhibition [49, 50], and is a degradation product of the fungicide 2-(thiocyanomethylthio)benzothiazole [51]. For these reasons, 2-methylsulfanyl-1,3-benzothiazole is being detected in municipal wastewater or indoor air [52, 53]. When comparing the results with our previous study on zebra finch preen oil and feathers, only one substance was identified as a potential sex signature candidate in both studies: namely, pentan-1-ol [31]. However, whereas in preen oil pentan-1-ol was only detected in female samples [31], the whole body odour samples showed the presence of pentan-1-ol in female Tenax® TA samples as well as in male activated charcoal samples. Follow-up studies are planned to further determine the concentrations of compounds suggested to occur in only one sex in this and our previous study [31] via targeted quantification in samples obtained from individual birds, using a higher number of samples. For full quantitative approaches, a validation of the described method should be performed that includes determination of the limit of detection, limit of quantitation, accuracy and precision, aspects that were beyond the scope of the present study.

## Conclusions

In the present study, whole body odour samples of zebra finches (*Taeniopygia castanotis*) have been sampled by applying an open loop system and using activated charcoal and Tenax® TA as sorbents. The aims were to characterize the volatilome and also to compare the two sorbents. In total, we identified 104 compounds, of which 12 have not been described in birds before. Qualitative and quantitative differences between the two sorbent types were detected. Nonetheless, most of the identified substances were in common across the two sorbent types, with some substances being present only in one adsorbent type. Semi-quantitative evaluation of the dataset showed that higher relative peak areas were detected for four carboxylic acids and lower relative peak areas for propanoic acid, DEHP and four alkanes in Tenax ® TA compared to activated charcoal. We conclude that both sorbents can be applied for the sampling of birds. Moreover, we determined potential candidate substances for a chemical sex signature by comparing occurrence of the substances in samples from male and female birds.

## Supplementary Information

Below is the link to the electronic supplementary material.

Supplementary file1 (DOCX 109 KB)

## Declarations

All procedures with the zebra finches were approved by the German authorities (LANUV license number AZ 81–02.04.2021.A432) and conducted according to the guidelines for animal experiments by LANUV. Samples were collected at the Department of Behavioural Ecology, Bielefeld University.
