# Supplementary material for: Analytical characterization of volatiles present in the whole body odour of zebra finches
Source: Anal Bioanal Chem. 2024 Aug 9;416(24):5335–49. doi: 10.1007/s00216-024-05466-8 (PMC11416363; doi:10.1007/s00216-024-05466-8)
Supplement: Supplementary file 1 — Supplementary file1 (DOCX 109 KB) [file 216_2024_5466_MOESM1_ESM.docx]

Supplementary material

Chemicals

Dichloromethane, ethoxyethane, methanol and anhydrous sodium sulfate were purchased from VWR Chemicals (Radnor, PA, USA). Chloroform was bought from Carl Roth GmbH + Co. KG (Karlsruhe, Germany) and hexane was purchased from Sigma-Aldrich (Steinheim, Germany). Dichloromethane and ethoxyethane was freshly distilled before usage. Glass thermo desorption tubes (6.35 mm x 89 mm) filled with silanized glass wool (Sigma-Aldrich, Steinheim, Germany) and porous polymer 2,6-diphenyl-p-phenylene oxide (in the following Tenax® TA 60/80) (200 mg), and Precision Charcoal Filters filled with 1.5 mg charcoal (Brechbühler AG, Schlieren, Switzerland) were used for the adsorption experiments. The following reference compounds were used for comparison: acetic acid (Aldrich, Steinheim, Germany, > 99 % purity), benzonitrile (Sigma-Aldrich, Steinheim, Germany, 99 % purity), (*Z*)-2-butyloct-2-enal (Sigma-Aldrich, Steinheim, Germany, ≥ 95 % purity), 4-butylphenol (ABCR, Karlsruhe, Germany, 98 % purity), butyl prop-2-enoate (in the following butyl acrylate, Sigma-Aldrich, Steinheim, Germany, 99 % purity), benzaldehyde (Fluka, Steinheim, Germany, 99 % purity), 1,3-benzothiazole (Aldrich, Steinheim, Germany, 96 % purity), bis(2-ethylhexyl) benzene-1,2-dicarboxylate (in the following DEHP, Sigma-Aldrich, Steinheim, Germany, reference material), butanoic acid (Fluka, Steinheim, Germany, > 99.5 % purity), butan-1-ol (Aldrich, Steinheim, Germany, 99.9 % purity), (2E,4E)-deca-2,4-dienal (in the following: (E,E)-2,4-decadienal, Fluka, Steinheim, Germany, 85 % purity), decanal (Sigma, purity unknown), decane (Fluka, Steinheim, Germany, ≥ 98 % purity), decanoic acid (Aldrich, Steinheim, Germany, 98 % purity), decan-1-ol (Sigma-Aldrich, Steinheim, Germany, 99 % purity), (4S,4aS,8aR)-4,8a-dimethyl-1,2,3,4,5,6,7,8-octahydronaphthalen-4a-ol (in the following geosmin, Aldrich, Steinheim, Germany, 98 % purity), 2,2-dimethyl-3-methylidenebicyclo[2.2.1]heptane (in the following camphene, Supelco, Bellefonte, USA, ≥ 96 % purity), diphenylmethanone (Aldrich, Steinheim, Germany, ≥ 99 %), 2,4-di*tert*-butylphenol (Sigma-Aldrich, Steinheim, Germany, 99 % purity), docosane (Fluka, Steinheim, Germany, > 99.5 % purity), docosan-1-ol (Sigma-Aldrich, Steinheim, Germany, 98 % purity), dodecanal (Aldrich, Steinheim, Germany, 92 % purity), dodecane (Fluka, Steinheim, Germany, > 98 % purity), dodecanoic acid (Aldrich, Steinheim, Germany, 98 % purity), dodecan-1-ol (Fluka, Steinheim, Germany, 98 % purity), 2-(2-ethoxyethoxy)ethanol (Sigma-Aldrich, Steinheim, Germany, ≥ 99 % purity), 2-ethylhexan-1-ol (Fluka, Steinheim, Germany, > 99 % purity), 1-ethyl-2-methylbenzene (ABCR, Karlsruhe, Germany, 98 % purity), 1-ethyl-3-methylbenzene (Sigma-Aldrich, Steinheim, Germany, 99 % purity), 4-ethyloctanoic acid (Aldrich, Steinheim, Germany, 98 % purity), furan-2-carbaldehyde (Sigma-Aldrich, Steinheim, Germany, 98 % purity), henicosane (Fluka, Steinheim, Germany, ≥ 99.5 % purity), heptacosane (Fluka, Steinheim, Germany, > 99.5 % purity), heptanal (Sigma-Aldrich, Steinheim, Germany, >92 % purity), heptane (Fluka, Steinheim, Germany, ≥ 99 % purity), heptanoic acid (Aldrich, Steinheim, Germany, 99 % purity), heptan-1-ol (Sigma-Aldrich, Steinheim, Germany, 98 % purity), heptan-2-one (Sigma-Aldrich, Steinheim, Germany, 99 % purity), hexacosane (Fluka, Steinheim, Germany, > 99.5 % purity), hexadecane (Fluka, Steinheim, Germany, purity unknown), hexadecanoic acid (Sigma-Aldrich, Steinheim, Germany, 99 % purity), propan-2-yl hexadecanoate (> 90 % purity, Sigma-Aldrich, Steinheim, Germany), hexadecan-1-ol (Sigma-Aldrich, Steinheim, Germany, 99 % purity), hexanal (Aldrich, Steinheim, Germany, 98 % purity), hexanoic acid (Aldrich, Steinheim, Germany, 99.5 % purity), hexan-1-ol (Fluka, Steinheim, Germany, ≥ 99 % purity), (*Z*)-hex-3-enal (50 % purity, SAFC, Steinheim, Germany), 4-hydroxy-3-methoxybenzaldehyde (in the following vanillin, ABCR, Karlsruhe, Germany, 99 % purity), icosane (Fluka, Steinheim, Germany, ≥ 99 % purity), 2-methoxyphenol (Aldrich, Steinheim, Germany, 98 % purity), 2-methylbutanoic acid (Aldrich, Steinheim, Germany, 98 % purity), 3-methylbutanoic acid (Aldrich, Steinheim, Germany, 99 % purity), methyl hexadecanoate (Sigma, Steinheim, Germany, ≥ 99 % purity), 4-methylnonanoic acid (Aldrich, Steinheim, Germany, 97 % purity), methyl octanoate (Aldrich, Steinheim, Germany, 99 % purity), methyl octadecanoate (Sigma, Steinheim, Germany, 99 % purity), 1-(3-methylphenyl)ethanone (Aldrich, Steinheim, Germany, 98 % purity), 1-(4-methylphenyl)ethanone (Fluka, Steinheim, Germany, 96 % purity), 2-methylpropanal (Aldrich, Steinheim, Germany, 99.5 % purity), 2-methylpropanoic acid (SAFC, Steinheim, Germany, 99 % purity), 1-methyl-3-propan-2-ylbenzene (in the following m-cymene, TCI Europe, Eschborn, Germany, 99 % purity), 1-methyl-4-propan-2-ylbenzene (in the following p-cymene, Fluka, Steinheim, Germany, 99.5 % purity), 2-methyl-5-propan-2-ylcyclohexa-1,3-diene (in the following α-phellandrene, SAFC, Steinheim, Germany, purity unknown), 1-methyl-4-prop-1-en-2-ylcyclohexene (in the following dipentene, Aldrich, Steinheim, Germany, technical grade), 2-methylsulfanyl-1,3-benzothiazole (Aldrich, Steinheim, Germany, 97 % purity), nonacosane (Fluka, Steinheim, Germany, > 99 % purity), (2*E*,4*E*)-nona-2,4-dienal (in the following (*E,E*)-2,4-nonadienal, Aldrich, Steinheim, Germany, 85 % purity), nonanal (Fluka, Steinheim, Germany, 95 % purity), nonane (Fluka, Steinheim, Germany, ≥ 99 % purity), nonanoic acid (Sigma, Steinheim, Germany, 97 % purity), nonan-1-ol (Sigma-Aldrich, Steinheim, Germany, > 98 % purity), (*E*)-non-2-enal (Aldrich, Steinheim, Germany, 97 % purity), (*Z*)-non-2-enal (Aldrich, Steinheim, Germany, > 90 % purity), octacosane (Fluka, Steinheim, Germany, purity unknown), octadecane (Fluka, Steinheim, Germany, purity unknown), octadecanoic acid (Alfa Aesar, Karlsruhe, Germany, 98 % purity), octadecan-1-ol (Sigma-Aldrich, Steinheim, Germany, purity unknown), (Z)-octadec-9-enoic acid (in the following oleic acid, Fluka, Steinheim, Germany, 99 % purity), octanal (Aldrich, Steinheim, Germany, 99 % purity), octanoic acid (SAFC, Darmstadt, Germany, 98 % purity), octan-1-ol (Fluka, Steinheim, Germany, > 99.5 % purity), oct-1-en-3-one (Aldrich, Steinheim, Germany, 96 % purity), 1-octoxyoctane (in the following dioctyl ether, Sigma-Aldrich, Steinheim, Germany, 99 % purity), oxolan-2-one (Sigma-Aldrich, Steinheim, Germany, > 99 % purity), pentacosane (Fluka, Steinheim, Germany, ≥ 99.5 % purity), pentadecan-1-ol (Acros Organics, Steinheim, Germany, 99 % purity), pentanoic acid (Fluka, Steinheim, Germany, 99 % purity), pentan-1-ol (Sigma-Aldrich, Steinheim, Germany, > 99 % purity), 3-(3-pentyloxiran-2-yl)prop-2-enal (in the following trans-4,5-epoxy-(*E*)-2-decenal, AromaLab, Freising, Germany, 97 % purity), 5-pentyloxolan-2-one (in the following *y*-nonalactone, Aldrich, Steinheim, Germany, > 98 % purity), phenol (Fluka, Steinheim, Germany, 99 % purity), 1-phenylethanone (SAFC, Steinheim, Germany, 98 % purity), phenylmethanol (Alfa Aesar, Karlsruhe, Germany, 99 % purity), (*E*)-3-phenylprop-2-enal (in the following (*E*)-cinnamaldehyde, Aldrich, Steinheim, Germany, 99 % purity), propanoic acid (Riedel-de-Haen, Seelze, Germany, 99 % purity), propan-2-yl hexadecanoate (in the following isopropyl palmitate, concentration and supplier unknown), propan-2-yl tetradecanoate (in the following isopropyl myristate, Sigma-Aldrich, Steinheim, Germany, 98 % purity), tetracosane (Fluka, Steinheim, Germany, > 99.5 % purity), tetradecane (Fluka, Steinheim, Germany, purity unknown), tetradecanoic acid (Fluka, Steinheim, Germany, 98 % purity), tetradecan-1-ol (Acros Organics, Steinheim, Germany, 99 % purity), triacontane (Alfa Aesar, Karlsruhe, Germany, > 98 % purity), tricosane (Fluka, Steinheim, Germany, > 99.5 % purity), tridecane (Fluka, Steinheim, Germany, ≥ 99 % purity), 2,6,6-trimethylbicyclo[3.1.1]hept-2-ene (in the following α-pinen, Aldrich, Steinheim, Germany, 97 % purity), 3,7,7-trimethylbicyclo[4.1.0]hept-3-ene (in the following 3-carene, SAFC, Steinheim, Germany, 90 % purity), undecanal (Aldrich, Steinheim, Germany, 97 % purity), undecane (Aldrich, Steinheim, Germany, ≥ 99 % purity), 1,2-xylene (Sigma-Aldrich, Steinheim, Germany, 99.5 % purity), 1,3-xylene (Sigma-Aldrich, Steinheim, Germany, 99.5 % purity) and 1,4-xylene (Sigma-Aldrich, Steinheim, Germany, 99.5 % purity). For the determination of retention indices, a diluted homologous series of alkanes ranging from hexane to triacontane (Fluka and Sigma-Aldrich, Steinheim, Germany) were applied.

Table S1: Odour-active substances in zebra finch whole body odour samples from Table 1. The table shows the identified substances together with their retention indices (RI) on a DB-FFAP and a DB-5 column, their CAS-number, their odor quality and their OD-factors. Substances identified with all identification criteria are marked in bold. Substances and unknown compounds that occurred in odor dilution (OD) factors < 2 are not shown. Substances of potential exogenous origin are marked with a superscripted B because they were also found in the blank sample.

| No. | Substance | CAS-No. | RI-DB FFAP | RI DB-5 | Odor quality | 10BC1 | 10BT1 | 10C | 10T | previously identified? |
| --- | --- | --- | --- | --- | --- | --- | --- | --- | --- | --- |
| 1 | unknown | - | 1655 | - | green, fruity, fatty | 4 |  |  |  |  |
| 2 | oct-1-en-3-one | 4312-99-6 | 1291 | 979 | mushroom-like |  |  | 8^1^ | 1^1^ |  |
| 3 | acetic acid^B^ | 64-19-7 | 1438 | s.d. | vinegar-like | MS^5^ | MS^5^ | 4^1^, MS^5^ | 2^1^, MS^5^ | y |
| 4 | unknown | - | 1450 | - | cardboard-like |  |  |  | 4 |  |
| 5 | (*Z*)-non-2-enal | 60784-31-8 | 1494 | 1145 | fatty, soapy, cucumber-like |  |  | 4^1^ | 16^2^ |  |
| 6 | **benz-aldehyde**^B^ | 100-52-7 | 1515 | 967 | bitter almond-like, almond-like | MS^4^ | MS^4^ | 8^1^, MS^4^ | MS^4^ | y |
| 7 | (*E*)-non-2-enal | 18829-56-6 | 1533 | 1160 | fatty, cucumber-like, cardboard-like |  |  | 4^1^ |  |  |
| 8 | unknown | - | 1562 | - | cardboard-like |  | 256 |  |  |  |
| 9 | unknown | - | 1600 | - | green, fatty |  |  | 8 |  |  |
| 10 | **butanoic acid**^B^ | 107-92-6 | 1618 | 804 | cheesy, sweaty | MS^5^ |  | 4^2^, MS^4^ | MS^5^ | y |
| 11 | 3-methylbutanoic acid/ 2-methylbutanoic acid* | 503-74-2/ 116-53-0 | 1653/ 1655 | 861/ 868 | cheesy/ apple-like, fruity |  |  | 4^2^, MS^5^ | 16384^1^ | y |
| 12 | (*Z*)-2-butyloct-2-enal^B^ | 99915-14-7 | 1663 | 1373 | fruity |  | 4^1^ | 8^2^ | 16384^2^ |  |
| 13 | (*E,E*)-2,4-nonadienal | 5910-87-2 | 1690 | 1213 | fatty, nutty |  |  | 32^2^ | 1^1^ |  |
| 14 | pentanoic acid^B^ | 109-52-4 | 1725 | 888 | fruity, sweaty, pungent | MS^5^ | MS^5^ | 1^2^, MS^5^ | MS^5^ | y |
| 15 | (*E,E*)-2,4-decadienal | 25152-84-5 | 1801 | 1327 | fatty, deep-fried |  |  | 1^1^ | 4^1^ |  |
| 16 | geosmin | 19700-21-1 | 1806 | 1421 | earthy, mouldy |  |  | 16^1^ |  |  |
| 17 | 2-methoxyphenol | 90-05-1 | 1846 | 1089 | smoky, smoked ham-like |  |  | 1^2^ | 4^2^ |  |
| 18 | **1,3-benzothiazole**^B^ | 95-16-9 | 1937 | 1227 | rubber-like, car tire-like | MS^4^ | MS^4^ | 128^3^, MS^4^ | MS^4^ |  |
| 19 | **2-methylsulfanyl-1,3-benzothiazole**^B^ | 615-22-5 | 1947 | 1235 | medicinal, smoky, phenolic | MS^6^ |  | GC-GC-MS/O, MS^4^ |  |  |
| 20 | unknown | - | 1961 | - | cardboard-like |  | 256 |  |  |  |
| 21 | unknown | - | 1975 | - | car tyre-like, burnt |  |  |  | 16384 |  |
| 22 | trans-4,5-epoxy-(*E*)-2-decenal | 134454-31-2 | 1991 | 1375 | metallic |  |  | 1^2^ | 8^2^ |  |
| 23 | **ɣ-nonalactone** | 104-61-0 | 2018 | 1360 | coconut-like |  |  | 16^2^ | 2^1^, MS^4^ | y |
| 24 | unknown | - | 2070 | - | mouldy, horse stable-like |  |  |  | 4 |  |
| 25 | 4-ethyloctanoic acid/ 4-methylnonanoic acid* | 16493-80-4/ 54947-74-9 | 2187/ 2198 | 1322/ 1328 | goat-like/ cardboard-like, plastic-like |  |  | 32^1^ | 16^1^ | y |
| 26 | unknown | - | 2194 | - | cardboard-like, mouldy, cheesy | 8 |  |  |  |  |
| 27 | decanoic acid^B^ | 334-48-5 | 2267 | 1371 | coriander-like, plastic-like, soapy | MS^5^ |  | 4^2^, MS^5^ | 4^1^, MS^5^ | y |
| 28 | unknown | - | 2318 | - | fatty, cardboard-like |  |  | 4 | 1 |  |
| 29 | unknown | - | 2350 | - | fatty |  |  |  | 4 |  |
| 30 | unknown |  | 2353 | - | burnt, car tire-like |  | 8 |  |  |  |
| 31 | unknown | - | 2421 | - | citrus-like, coriander-like, waxy |  |  |  | 8 |  |
| 32 | unknown | - | 2444 | - | eukalyptus-like, coriander-like |  |  | 16 |  |  |
| 33 | dodecanoic acid^B^ | 143-07-7 | 2473 | 1571 | waxy, soapy | MS^5^ | MS^5^ | 8^1^, MS^5^ | MS^5^ | y |
| 34 | unknown | - | 2490 | - | vanilla-like |  | 4 |  |  |  |
| 35 | unknown | - | 2521 | - | waxy |  |  |  | 4 |  |
| 36 | vanillin | 121-33-5 | 2564 | 1399 | vanilla-like, sweet |  | 8^1^ | 8^1^ |  |  |
| 37 | unknown | - | 2581 | - | soapy, coriander-like |  |  | 16 |  |  |
| 38 | unknown | - | 2589 | - | vanilla-like, cinammon-like, green |  |  |  | 4 |  |
| 39 | unknown | - | 2611 | - | vanilla-like |  |  |  | 16 |  |
| 40 | unknown | - | 2633 | - | cheesy, mouldy |  |  | 4 |  |  |
| 41 | unknown | - | 2674 | - | cheesy, honey-like |  |  |  | 16 |  |
| 42 | unknown | - | 2875 | - | cardboard-like, cheesy |  | 4 |  |  |  |
| 43 | unknown | - | 2935 | - | vanilla-like |  | 16 |  |  |  |

^1^ tentatively identified by comparison of RI & odor quality on DB-FFAP column with a reference compound; ^2^ tentatively identified by comparison of RI & odor quality on two columns (DB-FFAP, DB-5) with a reference compound; ^3^ Identified by comparison of RI, odor quality and mass spectra on two different columns (DB-FFAP, DB-5) with a reference compound; ^4^ Identified by comparison of RI & mass spectra on two columns (DB-FFAP, DB-5) with a reference compound; ^5^ tentatively identified by comparison of RI & mass spectrum on DB-FFAP column with a reference compound; ^6^ tentatively identified by comparison of RI & mass spectrum on DB-5 column with a reference compound; * Coelution of two compounds

Table S2: Further identified volatile compounds in zebra finch whole body odour samples via GC-MS. The table shows the further identified volatile compounds together with their retention indices (RI) on a DB-FFAP and a DB-5 column and their CAS-number. For sample types see Table 1 and 2 in the main manuscript. Substances that occurred in < 2 samples of each sample type are not shown. The 10 highest peaks were screened for unknown compounds and are listed in the table if they were detected in at least two samples of each sample type. Substances of potential exogenous origin are marked with a superscripted B because they were also found in the blank sample. * = Coelution of two compounds.

| No. | Name | CAS | RI FFAP | RI DB5 | 10BC1 | 10BC2 | 10BC3 | 10C | 10CF1 | 10CF2 | 10CF3 | 10CM1 | 10CM2 | 10CM3 | 10BT1 | 10BT2 | 10T | 10TF1 | 10TF2 | 10TF3 | 10TM1 | 10TM2 | 10TM3 |
| --- | --- | --- | --- | --- | --- | --- | --- | --- | --- | --- | --- | --- | --- | --- | --- | --- | --- | --- | --- | --- | --- | --- | --- |
| 1 | heptane^B^ | 142-82-5 | 700 | 700 |  |  |  |  |  |  |  |  |  |  |  | 3 |  |  |  | 3 |  |  | 3 |
| 2 | α-pinene^B^ | 80-56-8 | 806 | 937 |  | 3 | 3 | 3 | 3 | 3 |  | 3 | 3 | 3 | 3 | 3 | 3 | 3 | 3 | 3 | 3 | 3 | 3 |
| 3 | nonane^B^ | 111-84-2 | 900 | 900 |  | 3 |  | 3 | 3 |  |  | 3 | 3 |  |  |  |  |  |  |  |  |  |  |
| 4 | decane^B^ | 124-18-5 | 1000 | 1000 |  | 3 |  | 3 | 3 |  |  | 3 | 3 |  |  | 3 |  | 3 | 3 | 3 | 3 |  | 3 |
| 5 | 4-butylphenol^B^ | 1638-22-8 | 1033 | 1361 | 2 | 2 |  | 2 | 2 |  |  | 2 | 2 |  |  |  |  |  |  |  |  |  |  |
| 6 | camphene | 79-92-5 | 1067 | n.a. |  |  |  |  | 2 |  |  |  | 2 |  |  |  |  |  |  |  |  |  |  |
| 7 | hexanal^B^ | 66-25-1 | 1080 | 800 |  |  | 2 | 1 | 2 | 2 | 2 | 2 | 2 |  | 2 | 1 |  | 1 | 1 | 1 | 1 | 1 | 1 |
| 8 | unknown^B^ | 1120-21-4 | 1089 | n.a. |  | 2 |  |  | 2 |  |  |  | 2 |  |  |  |  |  |  |  |  |  |  |
| 9 | undecane^B^ | 106-42-3/ 108-38-3 | 1100 | 1100 |  | 3 |  |  | 3 |  |  | 3 | 3 |  |  | 3 |  | 3 |  |  | 3 | 3 |  |
| 10 | 1,4-xylene/1,3-xylene^B*^ | 13466-78-9 | 1130 | 877 | 1 | 1 | 1 | 1 | 1 | 1 | 1 | 1 | 1 | 1 | 1 | 1 | 2 | 1 | 1 | 1 | 2 | 2 | 1 |
| 11 | 3-carene^B^ | 71-36-3 | 1140 | n.a. |  |  |  |  | 2 |  |  |  |  |  | 2 |  | 2 | 2 |  | 2 | 2 | 2 |  |
| 12 | butan-1-ol^B^ | 99-83-2 | 1141 | s.d. | 2 | 2 | 2 | 2 |  | 2 | 2 |  | 2 | 2 |  | 2 |  |  | 2 | 2 |  |  | 2 |
| 13 | α-phellandrene | 110-43-0 | 1141 | n.a. |  |  |  |  |  |  |  |  |  |  |  |  |  | 2 |  |  |  | 2 |  |
| 14 | heptan-2-one^B^ | 141-32-2 | 1177 | 892 |  |  |  | 2 |  |  |  |  |  |  |  | 1 |  |  | 2 | 1 |  |  | 2 |
| 15 | butyl acrylate | 95-47-6 | 1178 | 900 |  |  |  | 3 |  |  |  |  | 2 |  |  |  |  |  |  |  |  |  |  |
| 16 | 1,2-xylene^B^ | 111-71-7 | 1179 | 899 | 2 | 2 | 2 | 2 | 2 |  | 3 | 2 | 2 | 3 |  |  |  | 2 |  |  | 3 | 3 |  |
| 17 | heptanal^B^ |  | 1179 | 906 |  |  |  |  | 3 | 3 | 3 | 3 |  | 3 | 3 | 3 |  | 3 | 3 | 3 | 3 | 3 | 3 |
| 18 | dipentene^B^ | 138-86-3 | 1192 | 1034 | 2 | 1 | 1 | 1 | 1 | 2 | 2 | 1 | 1 | 2 |  | 2 | 2 | 2 | 2 | 2 |  |  | 2 |
| 19 | dodecane^B^ | 112-40-3 | 1200 | 1200 |  | 3 | 3 | 3 | 3 | 3 | 3 | 3 |  | 3 | 3 | 3 | 3 | 3 | 3 | 3 | 3 | 3 | 3 |
| 20 | 1-ethyl-3-methylbenzene^B^ | 620-14-4 | 1219 | 963 |  | 3 |  | 1 | 2 |  |  |  |  |  |  |  |  |  |  |  |  |  |  |
| 21 | pentan-1-ol^B^ | 71-41-0 | 1236 | 776 | 2 | 2 |  | 2 |  |  |  |  | 2 | 3 |  |  |  |  | 2 | 1 |  |  |  |
| 22 | 1-ethyl-2-methylbenzene^B^ | 611-14-3 | 1257 | 980 | 2 | 2 | 2 | 1 | 1 | 2 |  | 1 | 2 | 2 |  | 2 |  | 1 |  | 2 |  |  | 2 |
| 23 | *p*-cymene/m-cymene^B*^ | 99-87-6 | 1262 | 1027 |  | 3 |  | 2 | 1 | 2 | 2 | 1 | 1 |  | 3 |  | 2 | 1 |  |  | 3 | 3 |  |
| 24 | octanal^B^ | 124-13-0 | 1280 | 1002 |  |  | 2 |  | 1 |  |  | 1 |  | 2 |  | 1 | 2 | 1 |  | 2 | 3 | 3 | 1 |
| 25 | tridecane^B^ | 629-50-5 | 1300 | 1300 |  | 3 |  |  |  |  |  |  |  | 3 | 3 | 3 |  | 3 | 3 | 3 | 3 | 3 | 3 |
| 26 | hexan-1-ol^B^ | 111-27-3 | 1336 | 872 | 2 | 2 | 1 | 1 | 2 | 1 | 1 | 2 | 2 | 1 |  | 2 |  |  |  | 3 |  |  | 1 |
| 27 | nonanal^B^ | 124-19-6 | 1381 | 1106 | 2 | 1 | 1 | 2 | 1 | 1 | 1 | 1 | 1 | 1 | 1 | 1 | 1 | 1 | 2 | 1 | 1 | 1 | 1 |
| 28 | tetradecane^B^ | 629-59-4 | 1400 | 1400 |  | 3 |  | 3 |  |  |  |  |  | 3 | 3 | 3 | 3 | 3 |  | 3 | 3 | 3 | 3 |
| 29 | acetic acid^B^ | 64-19-7 | 1438 | s.d. | 2 | 2 | 2 | 2 | 2 | 2 | 2 | 2 | 2 | 2 | 2 | 2 | 2 | 2 | 2 | 2 | 2 | 2 | 2 |
| 30 | heptan-1-ol^B^ | 111-70-6 | 1444 | 972 |  |  |  |  | 2 |  |  |  |  | 2 |  | 3 |  |  |  | 3 |  |  | 3 |
| 31 | furan-2-carbaldehyde^B^ | 98-01-1 | 1465 | 838 | 2 | 2 | 2 | 1 | 1 | 2 | 2 | 1 | 1 | 2 |  | 3 |  |  |  | 3 |  |  | 3 |
| 32 | 2-ethylhexan-1-ol^B^ | 104-76-7 | 1468 | 1030 | 2 | 1 | 1 | 1 | 1 | 1 | 1 | 1 | 1 | 1 | 1 | 1 | 1 | 1 | 1 | 1 | 1 | 1 | 1 |
| 33 | decanal^B^ | 112-31-2 | 1483 | 1208 |  |  | 1 |  | 3 | 3 | 1 | 3 |  | 1 |  | 1 |  | 2 | 1 | 1 | 2 | 2 | 1 |
| 34 | benzaldehyde^B^ | 100-52-7 | 1515 | 967 | 1 | 1 | 1 | 1 | 1 | 1 | 1 | 1 | 1 | 1 | 1 | 1 | 2 | 1 | 1 | 1 | 1 | 1 | 1 |
| 35 | propanoic acid^B^ | 79-09-4 | 1522 | 731 | 2 | 2 | 2 | 1 | 2 | 2 | 2 | 2 | 2 | 2 | 2 | 2 | 2 | 2 | 2 | 2 | 2 | 2 |  |
| 36 | octan-1-ol^B^ | 111-87-5 | 1545 | 1073 |  | 2 | 2 | 2 | 2 | 1 | 2 | 2 | 2 | 1 | 2 | 2 | 2 |  | 2 | 2 | 2 | 2 | 2 |
| 37 | 2-methylpropanoic acid/2-methylpropanal^B*^ | 79-31-2 | 1550 | 779 | 2 |  | 2 | 2 | 2 | 2 |  |  |  | 2 |  |  |  | 2 | 2 |  | 2 | 2 |  |
| 38 | undecanal^B^ | 112-44-7 | 1592 | 1309 |  |  |  |  |  |  |  |  |  |  |  | 3 |  |  | 2 |  |  |  | 1 |
| 39 | benzonitrile^B^ | 100-47-0 | 1597 | 989 | 2 |  |  | 1 | 1 |  |  | 1 |  | 1 |  | 1 |  |  | 1 | 1 |  |  | 1 |
| 40 | hexadecane^B^ | 544-76-3 | 1600 | 1600 |  |  |  |  |  |  |  |  |  |  |  | 3 | 3 | 3 |  | 3 | 3 | 3 | 3 |
| 41 | 1-phenylethanone^B^ | 98-86-2 | 1613 | 1070 | 3 |  | 2 | 3 | 3 | 1 | 2 | 3 | 3 | 1 | 3 | 1 |  | 3 | 1 | 1 | 3 | 3 | 1 |
| 42 | 2-(2-ethoxyethoxy)ethanol | 111-90-0 | 1618 | n.a. |  |  |  |  |  |  |  |  |  |  |  |  |  | 2 |  |  | 2 |  |  |
| 43 | butanoic acid^B^ | 107-92-6 | 1618 | 804 | 2 | 2 | 2 | 1 | 1 | 2 | 2 | 1 | 2 | 2 |  |  | 2 |  |  | 2 |  |  |  |
| 44 | oxolan-2-one^B^ | 96-48-0 | 1626 | 915 |  |  |  |  | 2 |  |  |  |  |  |  | 1 |  |  | 2 | 1 |  |  | 1 |
| 45 | nonan-1-ol^B^ | 143-08-8 | 1637 | 1173 |  |  |  | 2 |  |  |  |  |  | 2 |  | 2 | 3 |  | 2 |  |  | 2 |  |
| 46 | 3-methylbutanoic acid | 503-74-2 | 1653 | 861 |  |  |  |  |  | 2 |  |  |  | 2 |  |  |  |  |  |  |  |  |  |
| 47 | dodecanal^B^ | 112-54-9 | 1698 | 1411 |  |  |  |  |  |  |  |  |  |  |  | 3 |  | 2 | 2 | 3 | 3 |  | 3 |
| 48 | pentanoic acid^B^ | 109-52-4 | 1725 | 888 | 2 | 2 | 2 | 2 | 2 | 2 | 2 | 2 | 2 | 2 | 2 | 2 |  |  | 2 | 2 | 2 | 2 | 2 |
| 49 | unknown^B^ | - | 1734 | n.a. |  |  |  |  |  |  |  |  |  |  |  | 2 |  |  | 2 | 2 |  |  | 2 |
| 50 | decan-1-ol^B^ | 112-30-1 | 1740 | 1274 |  |  |  |  |  | 2 |  | 2 |  |  |  | 2 | 2 | 2 | 2 |  |  |  |  |
| 51 | 1-(3-methylphenyl)ethanone^B^ | 585-74-0 | 1745 | 1175 |  |  | 2 |  | 1 | 2 | 2 | 3 | 1 |  |  |  |  |  | 2 |  |  |  |  |
| 52 | dioctyl ether^B^ | 629-82-3 | 1745 | 1664 | 1 |  | 1 |  | 3 | 1 | 1 | 3 |  | 1 |  |  | 2 |  |  |  |  |  |  |
| 53 | 1-(4-methylphenyl)ethanone^B^ | 122-00-9 | 1766 | 1188 | 2 | 1 | 3 | 2 | 1 | 3 | 3 | 2 | 1 | 3 |  |  |  |  |  |  |  |  |  |
| 54 | unknown | - | 1774 | n.a. |  |  |  |  |  |  |  |  |  |  |  |  |  | 2 |  |  | 2 | 2 |  |
| 55 | octadecane^B^ | 593-45-3 | 1800 | 1800 |  |  |  |  |  |  |  |  |  |  |  | 3 | 3 | 3 | 3 | 3 |  | 3 | 3 |
| 56 | hexanoic acid^B^ | 142-62-1 | 1827 | 993 | 2 | 2 | 2 | 1 | 2 | 2 | 2 | 2 | 2 | 2 | 2 | 2 | 2 | 2 | 2 | 2 | 2 | 2 | 2 |
| 57 | unknown^B^ | - | 1854 | n.a. |  |  |  |  |  |  |  |  |  |  |  | 2 |  |  | 2 | 2 |  |  | 2 |
| 58 | phenylmethanol^B^ | 100-51-6 | 1866 | 1038 |  |  | 2 |  | 2 | 2 | 2 |  |  | 2 |  |  | 2 |  |  | 2 |  |  | 2 |
| 59 | unknown^B^ | - | 1894 | n.a. |  |  |  |  |  |  |  |  |  |  | 2 |  | 2 | 2 |  |  | 2 | 2 |  |
| 60 | heptanoic acid^B^ | 111-14-8 | 1934 | 1086 | 2 | 2 |  | 2 |  |  |  | 2 | 2 | 2 | 2 |  | 2 |  | 2 |  | 2 |  | 2 |
| 61 | 1,3-benzothiazole^B^ | 95-16-9 | 1937 | 1227 | 1 | 1 | 1 | 1 | 1 | 1 | 1 | 1 | 1 | 1 | 2 | 1 | 2 | 2 | 1 | 1 | 2 | 2 | 3 |
| 62 | dodecan-1-ol^B^ | 112-53-8 | 1946 | 1476 | 2 | 1 | 1 | 1 | 1 | 1 | 1 | 1 | 1 | 1 | 2 | 2 | 2 | 2 | 2 | 2 | 2 | 2 |  |
| 63 | icosane^B^ | 112-95-8 | 2000 | 2000 |  |  |  |  |  |  |  |  |  |  | 3 | 3 |  | 3 | 3 |  |  | 3 | 3 |
| 64 | phenol^B^ | 108-95-2 | 2000 | 981 |  |  | 1 |  | 1 | 1 | 1 | 1 | 1 | 1 | 1 | 1 |  | 1 | 1 | 1 | 1 | 1 | 1 |
| 65 | ɣ-nonalactone^B^ | 104-61-0 | 2018 | 1360 |  |  |  |  |  |  |  |  |  |  |  | 3 |  |  | 3 |  |  |  | 2 |
| 66 | isopropyl myristate^B^ | 110-27-0 | 2025 | 1823 | 1 |  |  |  |  | 3 |  | 3 |  | 3 | 3 | 3 |  |  |  |  | 3 | 3 | 3 |
| 67 | (*E*)-cinnamaldehyde | 104-55-2 | 2035 | 1276 |  |  |  |  | 3 | 3 |  |  |  |  |  |  |  |  |  |  |  |  |  |
| 68 | octanoic acid^B^ | 124-07-2 | 2052 | 1179 |  | 2 |  | 2 |  |  |  | 2 | 2 |  |  |  | 2 | 2 | 2 |  |  |  |  |
| 69 | (*Z*)-hex-3-enal^B^ | 6789-80-6 | 2071 | 806 | 2 |  |  |  |  | 2 |  |  |  | 2 | 2 |  |  | 2 |  |  |  |  |  |
| 70 | henicosane^B^ | 629-94-7 | 2100 | 2100 |  |  |  | 3 |  |  |  | 3 |  |  | 3 | 3 |  | 3 |  |  |  |  |  |
| 71 | nonanoic acid^B^ | 112-05-0 | 2149 | 1270 | 2 | 2 | 2 | 2 | 2 | 2 | 2 | 2 | 2 | 1 | 2 | 2 | 2 | 2 | 2 | 2 | 2 | 2 | 2 |
| 72 | tetradecan-1-ol^B^ | 112-72-1 | 2153 | 1679 | 2 | 2 | 1 | 1 |  | 1 | 1 | 1 | 2 | 1 | 2 | 2 | 2 | 2 |  |  |  |  | 2 |
| 73 | docosane^B^ | 629-97-0 | 2200 | 2200 |  | 3 | 3 | 3 |  | 3 | 3 | 3 |  | 3 | 3 | 3 |  | 3 | 3 | 3 |  | 3 | 3 |
| 74 | methyl hexadecanoate^B^ | 112-39-0 | 2203 | 1924 | 3 | 1 | 1 | 1 | 1 | 1 | 1 | 1 | 1 | 1 | 3 | 1 | 1 | 3 | 1 | 1 | 3 | 3 | 1 |
| 75 | isopropyl palmitate^B^ | 142-91-6 | 2230 | 2022 | 1 |  |  | 3 |  | 3 |  |  |  | 3 |  |  |  |  |  | 3 |  |  | 3 |
| 76 | unknown^B^ | - | 2246 | n.a. |  |  |  |  |  |  |  |  |  |  |  | 2 |  |  | 2 | 2 |  |  | 2 |
| 77 | pentadecan-1-ol | 629-76-5 | 2261 | 1782 |  |  |  |  |  |  |  |  |  |  |  |  | 3 |  |  |  | 3 |  |  |
| 78 | decanoic acid^B^ | 334-48-5 | 2267 | 1371 | 2 | 2 | 2 | 2 |  | 2 |  | 2 | 2 |  |  |  | 2 | 2 | 2 | 2 |  | 2 | 2 |
| 79 | unknown^B^ | - | 2270 | n.a. |  |  |  |  |  |  |  |  |  |  |  | 2 |  |  | 2 |  |  |  | 2 |
| 80 | tricosane^B^ | 638-67-5 | 2300 | 2300 |  | 3 | 3 |  |  | 3 | 3 | 3 |  | 3 | 3 | 3 |  | 3 | 3 | 3 |  | 3 | 3 |
| 81 | 2,4-di-*tert*-butylphenol^B^ | 96-76-4 | 2306 | 1509 |  |  |  |  |  |  |  |  |  |  | 1 | 3 | 1 | 1 | 3 | 3 | 1 | 1 | 3 |
| 82 | unknown | - | 2321 | n.a. |  |  |  |  |  |  |  |  |  |  |  |  |  | 2 |  |  | 2 | 2 |  |
| 83 | hexadecan-1-ol^B^ | 36653-82-4 | 2362 | 1896 | 2 | 2 | 2 |  | 2 | 2 | 2 | 2 | 2 | 2 |  |  | 2 |  |  | 2 |  |  | 2 |
| 84 | unknown | - | 2384 | n.a. |  |  |  |  |  | 2 |  |  |  | 2 |  |  |  |  |  |  |  |  |  |
| 85 | tetracosane^B^ | 646-31-1 | 2400 | 2400 |  | 3 | 3 | 3 |  | 3 | 3 | 3 |  | 3 | 3 |  |  | 3 |  |  | 3 | 3 |  |
| 86 | methyl octadecanoate^B^ | 112-61-8 | 2413 | 2126 | 1 | 2 | 2 | 1 | 1 | 1 | 1 |  | 1 | 3 | 3 | 1 | 1 | 3 | 3 | 3 | 1 | 3 | 3 |
| 87 | 2-methylsulfanyl-1,3-benzothiazole^B^ | 615-22-5 | 2437 | 1619 | 3 |  |  | 1 |  |  |  | 2 | 2 | 3 |  |  |  |  |  |  |  |  |  |
| 88 | unknown^B^ | - | 2460 | n.a. |  |  | 2 |  |  |  | 2 |  |  | 2 |  |  |  |  |  |  |  |  |  |
| 89 | dodecanoic acid^B^ | 143-07-7 | 2473 | 1571 | 2 | 2 |  | 2 |  | 2 |  |  | 2 | 2 | 2 | 2 | 2 | 2 | 2 | 2 | 2 | 2 | 2 |
| 90 | diphenylmethanone^B^ | 119-61-9 | 2477 | n.a. |  | 2 |  |  |  |  |  | 2 | 2 |  |  | 2 | 2 | 2 | 2 | 2 | 2 | 2 | 2 |
| 91 | pentacosane^B^ | 629-99-2 | 2500 | 2500 |  | 3 | 3 | 3 |  | 3 | 3 | 3 |  | 3 | 3 | 3 |  | 3 | 3 | 3 | 3 | 3 | 3 |
| 92 | octadecan-1-ol^B^ | 112-92-5 | 2577 | 2089 | 2 | 1 | 1 | 2 | 1 | 1 | 2 | 3 | 3 | 1 |  | 1 | 2 | 2 |  | 1 |  |  | 1 |
| 93 | hexacosane^B^ | 630-01-3 | 2600 | 2600 |  | 3 | 3 | 3 | 3 | 3 | 3 | 3 | 3 | 3 | 3 | 3 |  | 3 | 3 | 3 | 3 | 3 | 3 |
| 94 | unknown^B^ | - | 2643 | n.a. |  |  |  |  |  |  |  |  |  |  |  | 2 |  |  |  | 2 |  |  | 2 |
| 95 | unknown^B^ | - | 2658 | n.a. |  |  | 2 |  |  | 2 | 2 |  |  | 2 |  |  |  |  |  |  |  |  |  |
| 96 | tetradecanoic acid^B^ | 39525-69-4 | 2682 | 1761 | 2 |  | 2 | 2 |  | 2 | 2 | 2 |  | 2 | 2 |  | 2 | 2 |  |  | 2 | 2 | 2 |
| 97 | heptacosane^B^ | 593-49-7 | 2700 | 2700 |  |  | 3 | 3 |  | 3 | 3 | 3 |  | 3 | 3 | 3 |  | 3 | 3 | 3 | 3 | 3 | 3 |
| 98 | unknown^B^ | - | 2754 | n.a. |  |  | 2 |  |  | 2 | 2 |  |  | 2 |  |  |  |  |  |  |  |  |  |
| 99 | unknown^B^ | - | 2770 | n.a. |  |  |  |  |  |  |  |  |  |  |  | 2 |  |  | 2 | 2 |  |  | 2 |
| 100 | octacosane^B^ | 630-02-4 | 2800 | 2800 |  | 3 | 3 | 3 | 3 | 3 | 3 | 3 |  | 3 | 3 | 3 |  | 3 | 3 | 3 | 3 | 3 | 3 |
| 101 | unknown | - | 2817 | n.a. |  |  |  |  |  |  |  |  |  |  |  |  | 2 | 2 |  |  | 2 |  |  |
| 102 | unknown | - | 2852 | n.a. |  |  |  |  |  | 2 | 2 |  |  | 2 |  |  |  |  |  |  |  |  |  |
| 103 | hexadecanoic acid^B^ | 57-10-3 | 2895 | 1960 | 1 | 2 | 2 | 1 | 1 | 1 | 1 | 3 | 1 | 1 | 1 | 1 | 1 | 1 | 1 | 1 | 2 | 1 | 1 |
| 104 | nonacosane^B^ | 630-03-5 | 2900 | 2900 |  | 3 | 3 | 3 |  | 3 | 3 | 3 |  | 3 | 3 | 3 |  | 3 | 3 | 3 | 3 | 3 | 3 |
| 105 | docosan-1-ol^B^ | 661-19-8 | 2996 | 2497 | 2 |  | 2 |  |  | 1 | 2 |  |  | 2 |  |  | 2 |  |  |  |  |  |  |
| 106 | triacontane^B^ | 638-68-6 | 3000 | 3000 |  | 3 |  | 3 |  | 3 | 3 | 3 |  | 3 | 3 | 3 |  | 3 | 3 | 3 | 3 | 3 | 3 |
| 107 | octadecanoic acid^B^ | 57-11-4 | 3135 | 2162 | 2 |  |  | 2 |  |  |  |  |  |  |  |  | 1 | 2 | 3 | 3 | 2 | 1 |  |
| 108 | oleic acid | 112-80-1 | 3167 | 2137 |  |  |  | 2 |  |  |  |  |  |  |  |  | 2 | 2 |  |  |  |  |  |
| 109 | DEHP^B^ | 117-81-7 | 3201 | n.a. | 2 | 2 | 2 | 2 | 2 | 2 | 2 | 2 | 2 | 2 | 2 | 2 | 2 | 2 | 2 | 2 | 2 | 2 | 2 |

^1^ Identified by comparison of RI and mass spectrum with a reference compound on two columns (DB-FFAP, DB-5) via AMDIS; ^2^ tentatively identified by comparison of RI and mass spectrum with a reference compound on a DB-FFAP column via AMDIS, ^3^ tentatively identified by comparison of RI and mass spectrum with a reference compound on a DB-5 column via AMDIS, n.a. not available, s.d. elution within solvent delay.

Table S3: Average relative peak areas [*10^-3^] ± standard deviation obtained from semi quantification for female and male whole body odour. The table shows the substances together with their usage in ANOVA I/II, CAS-numbers and retention indices.

| ANOVA I/II | Substance | CAS-No. | RI DB-FFAP | RI DB-5 | Activated charcoal | | | Tenax® TA | | |
| --- | --- | --- | --- | --- | --- | --- | --- | --- | --- | --- |
|  |  |  |  |  | blank [10^-3^] | female [10^-3^] | male [10^-3^] | blank [10^-3^] | female [10^-3^] | male [10^-3^] |
| I&II | α-pinene | 80-56-8 | 806 | 937 | 2.8±1.8 | 4.9±5.6 | 6.9±0.9 | 9.4±1.8 | 9.9±7.0 | 12.4±1.6 |
| I&II | decane | 124-18-5 | 1000 | 1000 | 95.1±134.5 | 7.1±12.2 | 14.1±14.9 | 5.4±7.7 | 15.5±9.3 | 16.3±19.9 |
| I&II | hexanal | 66-25-1 | 1080 | 800 | 3.9±5.6 | 6.8±0.7 | 2.6±2.3 | 22.0±28.5 | 8.8±1.4 | 17.3±13.7 |
| I | undecane | 1120-21-4 | 1100 | 1100 | 74.1±104.8 | 8.6±14.9 | 18.8±16.7 | 8.9±12.6 | 46.0±79.7 | 118.3±109.2 |
| I&II | 1,4-/1,3-xylene* | 106-42-3/ 108-38-3 | 1130/ 1139 | 877/875 | 85.1±83.7 | 138.4±213.2 | 129.4±111.3 | 5.5±4.6 | 4.5±2.3 | 6.9±4.7 |
| I&II | butan-1-ol | 71-36-3 | 1141 | s.d. | 16.5±11.1 | 4.8±4.1 | 9.5±11.9 | 2.7±3.8 | 2.6±2.2 | 1.4±2.5 |
| I&II | 1,2-xylene | 95-47-6 | 1179 | 899 | 0.0±0.0 | 1.7±3.0 | 1.7±2.9 | 0.0±0.0 | 0.0±0.0 | 2.2±2.6 |
| I&II | heptanal | 111-71-7 | 1179 | 906 | 0.0±0.0 | 3.7±1.4 | 2.7±3.8 | 15.2±19.0 | 10.2±5.8 | 16.4±24.4 |
| I&II | dipentene | 138-86-3 | 1192 | 1034 | 12.4±5.7 | 9.7±7.9 | 16.6±8.3 | 1.5±2.1 | 9.3±11.1 | 1.7±2.9 |
| I&II | dodecane | 112-40-3 | 1200 | 1200 | 77.6±81.5 | 22.3±5.4 | 14.5±12.7 | 388.2±380.2 | 329.9±449.9 | 616.7±410.4 |
| I&II | 1-ethyl-2-methylbenzene | 611-14-3 | 1257 | 980 | 7.7±8.1 | 5.3±7.6 | 7.9±4.7 | 1.7±2.3 | 1.7±1.6 | 1.5±2.7 |
| I&II | *p*-/*m*-cymene* | 99-87-6/ 535-77-3 | 1262/ 1262 | 1027/ 1021 | 5.3±7.5 | 4.8±8.4 | 8.8±8.8 | 1.2±1.8 | 0.9±1.5 | 1.9±1.6 |
| I&II | octanal | 124-13-0 | 1280 | 1002 | 8.0±11.3 | 1.4±2.4 | 4.4±6.7 | 22.2±31.4 | 11.2±10.8 | 13.0±22.5 |
| II | tridecane | 629-50-5 | 1300 | 1300 | 67.6±95.6 | 0.0±0.0 | 5.5±9.6 | 209.4±148.7 | 160.4±183.9 | 297.1±138.0 |
| II | hexan-1-ol | 111-27-3 | 1336 | 872 | 13.6±2.4 | 12.5±6.8 | 12.5±0.3 | 0.4±0.6 | 0.0±0.0 | 0.8±1.5 |
| I&II | nonanal | 124-19-6 | 1381 | 1106 | 20.4±1.5 | 24.0±12.5 | 25.3±16.7 | 87.9±6.4 | 84.7±67.3 | 108.2±29.7 |
| I&II | acetic acid | 64-19-7 | 1438 | s.d. | 51.3±43.1 | 37.6±29.7 | 50.8±13.2 | 127.9±9.6 | 162.2±23.8 | 119.3±42.2 |
| II | furan-2-carbaldehyde | 98-01-1 | 1465 | 838 | 5.2±1.0 | 6.1±4.8 | 7.4±4.0 | 0.0±0.0 | 0.0±0.0 | 0.0±0.0 |
| I&II | 2-ethylhexan-1-ol | 104-76-7 | 1468 | 1030 | 209.4±12.1 | 188.4±19.6 | 195.4±34.7 | 561.7±547.5 | 451.2±528.7 | 823.6±547.6 |
| I&II | decanal | 112-31-2 | 1483 | 1208 | 2.3±3.3 | 2.3±4.0 | 2.4±4.1 | 25.3±35.8 | 21.6±10.8 | 43.0±38.3 |
| I&II | benzaldehyde | 100-52-7 | 1515 | 967 | 103.7±73.6 | 81.7±68.2 | 182.9±88.9 | 65.0±68.5 | 44.2±25.0 | 56.1±66.2 |
| I&II | propanoic acid | 79-09-4 | 1522 | 731 | 29.0±12.9 | 19.5±10.9 | 27.9±2.5 | 13.3±1.8 | 11.3±2.5 | 13.9±12.1 |
| I&II | octan-1-ol | 111-87-5 | 1545 | 1073 | 7.1±7.4 | 2.3±1.2 | 4.6±1.6 | 9.4±3.3 | 2.2±2.4 | 13.2±4.5 |
| I&II | 2-methylpropanoic acid/2-methylpropanal* | 79-31-2 | 1550/ 1561 | 779/n.a. | 0.9±1.3 | 1.8±1.7 | 1.2±2.1 | 0.0±0.0 | 0.9±0.8 | 2.0±1.7 |
| I&II | benzonitrile | 100-47-0 | 1597 | 989 | 0.0±0.0 | 1.1±2.0 | 1.7±1.6 | 7.6±10.7 | 4.7±4.1 | 4.8±8.4 |
| I | 1-phenylethanone | 98-86-2 | 1613 | 1070 | 4.1±5.8 | 5.9±6.4 | 3.7±6.4 | 76.7±108.5 | 46.8±41.0 | 49.0±84.9 |
| II | hexadecane | 544-76-3 | 1600 | 1600 | 0.0±0.0 | 0.0±0.0 | 0.0±0.0 | 28.3±40.0 | 32.0±34.9 | 73.9±47.8 |
| II | butanoic acid | 107-92-6 | 1618 | 804 | 27.1±14.4 | 17.5±9.1 | 23.5±0.2 | 0.0±0.0 | 1.1±2.0 | 0.0±0.0 |
| II | dodecanal | 112-54-9 | 1698 | 1411 | 0.0±0.0 | 0.0±0.0 | 5.7±6.0 | 0.0±0.0 | 0.0±0.0 | 0.0±0.0 |
| I&II | pentanoic acid | 109-52-4 | 1725 | 888 | 16.2±11.7 | 10.8±6.1 | 15.4±2.7 | 9.8±0.2 | 5.2±4.6 | 10.9±2.3 |
| II | 1-(4-methylphenyl)-ethanone | 122-00-9 | 1766 | 1188 | 24.6±34.8 | 6.9±12.0 | 0.0±0.0 | 0.0±0.0 | 15.6±18.6 | 0.0±0.0 |
| II | octadecane | 593-45-3 | 1800 | 1800 | 0.0±0.0 | 0.0±0.0 | 0.0±0.0 | 24.7±34.9 | 40.9±20.6 | 50.0±52.1 |
| I&II | hexanoic acid | 142-62-1 | 1827 | 993 | 34.1±12.1 | 30.7±16.0 | 34.0±10.4 | 32.9±0.5 | 37.7±6.4 | 51.8±15.1 |
| I | heptanoic acid | 111-14-8 | 1934 | 1086 | 6.9±9.7 | 0.0±0.0 | 6.7±2.5 | 1.7±2.3 | 4.9±8.6 | 3.0±2.7 |
| I&II | 1,3-benzothiazole | 95-16-9 | 1937 | 1227 | 46.3±38.0 | 61.0±27.9 | 150.9±70.9 | 10.2±2.3 | 12.1±2.7 | 7.5±7.0 |
| I&II | dodecan-1-ol | 112-53-8 | 1946 | 1476 | 58.7±1.0 | 70.0±49.6 | 39.6±16.7 | 56.4±5.5 | 34.7±9.7 | 19.2±18.5 |
| I&II | phenol | 108-95-2 | 2000 | 981 | 28.1±39.7 | 32.0±17.9 | 28.0±23.5 | 906.5±517.0 | 810.4±693.6 | 1219.6±565.6 |
| II | icosane | 112-95-8 | 2000 | 2000 | 0.0±0.0 | 0.0±0.0 | 0.0±0.0 | 191.4±94.2 | 61.7±58.6 | 58.5±52.1 |
| I | isopropyl myristate | 110-27-0 | 2025 | 1823 | 0.0±0.0 | 5.9±10.3 | 6.7±5.9 | 29.7±12.6 | 0.0±0.0 | 50.0±40.4 |
| I&II | nonanoic acid | 112-05-0 | 2149 | 1270 | 35.5±27.9 | 38.6±23.2 | 33.5±12.7 | 81.6±12.0 | 79.3±18.3 | 113.3±23.1 |
| II | tetradecan-1-ol | 112-72-1 | 2153 | 1679 | 48.6±5.9 | 45.2±39.4 | 282.2±421.5 | 32.2±26.3 | 5.1±8.8 | 32.6±56.5 |
| I&II | docosane | 629-97-0 | 2200 | 2200 | 220.8±170.8 | 63.4±75.9 | 137.6±122.0 | 1744.1±2117.8 | 248.2±44.6 | 160.2±138.7 |
| I&II | methyl hexadecanoate | 112-39-0 | 2203 | 1924 | 276.3±149.1 | 590.7±366.3 | 234.4±85.3 | 179.9±14.9 | 179.1±23.6 | 207.7±35.2 |
| I&II | decanoic acid | 334-48-5 | 2267 | 1371 | 13.2±4.5 | 1.7±3.0 | 5.4±5.9 | 0.0±0.0 | 12.3±7.6 | 15.3±14.0 |
| I&II | tricosane | 638-67-5 | 2300 | 2300 | 365.0±163.1 | 167.6±181.1 | 345.5±305.2 | 3612.9±4813.5 | 197.5±42.3 | 131.6±114.7 |
| II | 2,4-di-*tert*-butylphenol | 96-76-4 | 2306 | 1509 | 0.0±0.0 | 0.0±0.0 | 0.0±0.0 | 679.0±67.1 | 407.8±488.7 | 1209.4±484.7 |
| II | hexadecan-1-ol | 36653-82-4 | 2362 | 1896 | 217.9±17.0 | 325.5±231.8 | 171.8±83.6 | 0.0±0.0 | 13.9±24.0 | 47.7±82.7 |
| I&II | tetracosane | 646-31-1 | 2400 | 2400 | 448.5±14.0 | 409.8±398.7 | 663.3±577.4 | 4363.1±6170.4 | 145.2±251.5 | 161.9±184.1 |
| I&II | methyl octadecanoate | 112-61-8 | 2413 | 2126 | 0.0±0.0 | 157.5±193.5 | 67.9±68.5 | 62.6±48.2 | 69.3±25.1 | 71.0±25.3 |
| I&II | dodecanoic acid | 143-07-7 | 2473 | 1571 | 10.5±14.8 | 7.3±12.7 | 9.8±9.9 | 47.4±43.1 | 82.9±93.8 | 125.0±56.3 |
| II | diphenylmethanone | 119-61-9 | 2477 | n.a. | 12.2±17.2 | 0.0±0.0 | 3.9±4.8 | 22.9±32.4 | 24.7±3.5 | 31.9±25.3 |
| I&II | pentacosane | 629-99-2 | 2500 | 2500 | 561.4±40.3 | 509.0±563.4 | 857.0±746.4 | 6150.9±8137.3 | 347.7±113.2 | 377.5±249.3 |
| I&II | octadecan-1-ol | 112-92-5 | 2577 | 2089 | 109.3±33.7 | 170.2±115.0 | 47.1±81.5 | 22.6±31.9 | 27.0±25.2 | 28.1±48.6 |
| I&II | hexacosane | 630-01-3 | 2600 | 2600 | 590.3±58.2 | 719.6±734.7 | 1053.4±847.9 | 6827.3±8420.7 | 560.5±12.8 | 643.2±426.1 |
| I&II | tetradecanoic acid | 39525-69-4 | 2682 | 1761 | 24.0±33.9 | 37.1±49.6 | 20.3±28.2 | 17.1±24.2 | 49.2±85.3 | 179.7±116.9 |
| I&II | heptacosane | 593-49-7 | 2700 | 2700 | 279.8±395.7 | 844.8±947.5 | 1100.3±966.6 | 6542.4±8274.7 | 483.6±132.1 | 400.4±274.1 |
| I&II | octacosane | 630-02-4 | 2800 | 2800 | 448.4±43.9 | 816.9±844.3 | 952.6±832.9 | 6127.1±7664.1 | 411.9±18.1 | 281.1±153.5 |
| I&II | hexadecanoic acid | 57-10-3 | 2895 | 1960 | 516.1±251.4 | 467.2±337.9 | 291.2±161.7 | 1039.9±552.7 | 1286.5±419.7 | 1560.5±234.2 |
| I&II | nonacosane | 630-03-5 | 2900 | 2900 | 296.3±27.1 | 786.3±876.3 | 733.7±647.0 | 5051.2±6660.7 | 331.9±140.5 | 234.2±133.0 |
| I&II | triacontane | 638-68-6 | 3000 | 3000 | 250.9±354.8 | 594.4±661.7 | 490.7±434.7 | 4017.6±5149.3 | 357.5±174.4 | 384.5±388.9 |
| I&II | DEHP | 57-11-4 | 3135 | 2162 | 2347.8±968.6 | 3719.7±963.5 | 1709.6±392.9 | 142.7±184.3 | 163.4±20.5 | 146.1±62.9 |

Table S4: ANOVA I. Analysis of data with the independent factors substance (48), adsorbent (2) and sex (2) on the relative peak areas obtained from semi quantification. This data set was used to report the impact of adsorbent type (activated charcoal/Tenax® TA) as well as their interaction with the independent factor substance.

|  | df | df error | F | Sig. | partial η² |
| --- | --- | --- | --- | --- | --- |
| Substance | 1.375 | 2.749 | 30.104 | <.001 | 0.938 |
| Adsorbent | 1.000 | 2.000 | 2.812 | 0.236 | 0.584 |
| Sex | 1.000 | 2.000 | 0.002 | 0.969 | 0.001 |
| Substance*adsorbent | 1.650 | 3.299 | 22.785 | 0.012 | 0.919 |
| Substance*sex | 1.070 | 2.139 | 1.036 | 0.418 | 0.341 |
| Adsorbent*sex | 1.000 | 2.000 | 0.097 | 0.785 | 0.046 |
| Substance*adsorbent*sex | 1.096 | 2.193 | 1.070 | 0.412 | 0.349 |

Table S5: ANOVA II. Analysis of data with the independent factors substance (57), adsorbent (2) and sex (2) on the relative peak areas obtained from semi quantification. This data set was used to report the impact of sex (male/female) as well as their interaction with the independent factor substance.

|  | df | df error | F | Sig. | partial η² |
| --- | --- | --- | --- | --- | --- |
| Substance | 1.373 | 2.745 | 30.252 | <.001 | 0.938 |
| Adsorbent | 1.000 | 2.000 | 1.472 | 0.349 | 0.424 |
| Sex | 1.000 | 2.000 | 0.047 | 0.848 | 0.023 |
| Substance*adsorbent | 1.629 | 3.259 | 23.080 | 0.012 | 0.920 |
| Substance*sex | 1.157 | 2.315 | 1.065 | 0.414 | 0.347 |
| Adsorbent*sex | 1.000 | 2.000 | 0.135 | 0.748 | 0.063 |
| Substance*adsorbent*sex | 1.150 | 2.299 | 1.126 | 0.402 | 0.360 |
